# Linking anatomical diversity with functional life strategies in a liana community of central Africa

**DOI:** 10.1111/nph.70413

**Published:** 2025-08-04

**Authors:** Begüm Kaçamak, Maxime Réjou‐Méchain, Maël Grolleau, Jean‐Joël Loumeto, Grace Jopaul Loubota Panzou, Nick Rowe

**Affiliations:** ^1^ AMAP Univ. Montpellier, IRD, CNRS, CIRAD, INRAE 34000 Montpellier France; ^2^ Cirad UPR Forêts et Sociétés 34000 Montpellier France; ^3^ Laboratoire de Biodiversité, de Gestion des Ecosystèmes et de l'Environnement (LBGE), Faculté des Sciences et Techniques Université Marien Ngouabi BP69 Brazzaville Republic of the Congo; ^4^ Institut Supérieur des Sciences Géographiques, Environnementales, et de l'Aménagement (ISSGEA) Université DENIS SASSOU‐N'GUESSO BP1071 Kintélé Republic of the Congo

**Keywords:** anatomical diversity, cambial variants, Central Africa, ecological strategies, functional traits, liana ecology, vascular variants, woody vines

## Abstract

- Studying lianas involves unique challenges due to their high functional diversity. Despite their prominence in tropical forests, gaps in the understanding of anatomical organisation affect interpretations of their co‐existing functional strategies. Here, we investigated the roles of diverse vascular variants for better understanding liana ecology in northern Congo.
- We collected adult liana stems coexisting in an environmentally uniform area and acquired anatomical images of stem cross‐sections to characterise tissue patterns of vascular variants. We analysed anatomical diversity through functional traits to infer life‐history strategies, especially related to mechanical resilience and water use.
- We identified two main axes of trait variation: a trade‐off between hydraulic efficiency and safety, and a contrast between complex anatomical forms likely favouring flexibility against more simple forms favouring more rigid stems in early‐life strategies. While vascular variants showed relatively low intraspecific variability, they also displayed high developmental plasticity driven by ontogenetic variations and the local environment.
- This pioneering study establishes for the first time a connection between functional strategies and vascular variants, quantifying their diversity within a liana community. It represents a crucial step for understanding the complexity of lianas and their adaptive strategies, offering insights into their spatiotemporal distribution in diverse environments.

## Introduction

Lianas are an emblematic growth form of tropical forests, but also one of the most diverse plant forms (Chery *et al*., 2020b) resulting from the evolution of the lianoid species in > 130 angiosperm families (Gentry, 1991). Many lianas possess some of the most complex vascular organisations known in plants, a feature widely believed to be linked to the specific physiological, mechanical, and ecological constraints that have shaped diverse climbing life histories (Hegarty, 1991; Rowe & Speck, 2005; Angyalossy *et al*., 2015; Chery *et al*., 2020a). Due to its complexity, the diversity of vascular structures and their associated functions has rarely been explored at the community level, leaving the role of liana vascular diversity poorly understood. This complexity also makes it challenging to study and interpret the ecological implications of such diversity, including how their functional biology and environmental responses influence their distribution (Schnitzer & Bongers, 2002).

Lianas germinate on the ground and most exhibit a clear self‐supporting (upright) phase in the early stages of development. Following this, and just before or after attachment to a mechanical support, most species show a transition to a non‐self‐supporting (climbing) phase on host supports such as trees (Darwin, 1875; Putz, 1984; Putz & Holbrook, 1991; Caballé, 1998; Isnard & Silk, 2009). This profound change in development and associated functional traits has been characterized for numerous species and a wide diversity of climbing life histories (Putz & Mooney, 1991; Rowe & Speck, 1996, 2015; Rowe *et al*., 2006). However, it is challenging to study at the community level due to complex interactions between species and the environment and to the diversity of co‐existing strategies, especially in tropical forests. While standardized protocols have been proposed to measure liana stem diameters near ground level for large‐scale studies (Gerwing *et al*., 2006; Schnitzer *et al*., 2008), stem functional traits exhibit anatomical (e.g. complexity of wood organisation), physical (e.g. wood density), mechanical (e.g. stiff to flexible) and diverse outer shapes and profiles (e.g. circular, lobed and even ribbon‐shaped). All of these features are ontogenetically complex and vary from young to old stages of the liana. In particular, these traits can vary drastically from the self‐supporting to the non‐self‐supporting phase (Lahaye *et al*., 2005; Zhang *et al*., 2024). Therefore, understanding how liana anatomical properties vary across taxa and through their lifetime is crucial for unravelling the different life history strategies, ecological roles and distribution patterns of lianas in tropical forests.

During the early self‐supporting phase, many lianas show some similarities to young trees, treelets or shrubs, with centripetal wood development and centrifugal secondary phloem. The ‘juvenile wood’ in lianas is characterized by narrow, sparse or absent vessels, thick‐walled fibre cells, and limited, sometimes lignified axial parenchyma (Caballé, 1998; Angyalossy *et al*., 2012). Consequently, juvenile wood in lianas generally has high wood density and stiffness, providing mechanical support to self‐supporting stems (Fisher & Ewers, 1991; Rowe *et al*., 2004; Rowe & Speck, 2005). Some liana species exit the juvenile phase and begin adult development only after they attach to a support, rather than maturing according to a predetermined developmental trajectory (Rowe & Speck, 1996; Soffiatti *et al*., 2022). This shift from free‐standing to climbing involves a fundamental mechanical change from stiff to compliant stems, with mature lianas typically showing increased stem complexity, flexibility, and resilience and toughness against mechanical constraints (e.g. Obaton, 1960; Putz & Holbrook, 1991; Gallenmüller *et al*., 2001; Rowe & Speck, 2005). Lianas economize on structural costs per metre height by climbing supports and developing slender stems compared to trees. However, this ‘economy’ on materials makes them vulnerable to tree falls, swaying, wind action, falling branches, and other mechanical disturbances, which can break small‐diameter climbing stems if they have a brittle organisation (Ménard *et al*., 2013). As a result, many liana species have evolved anatomically complex woody forms during maturation, known under a range of terms including ‘anomalous secondary growth’ (Fahn, 1982), ‘vascular cambial variants’ (Carlquist, 1991), and more recently ‘vascular variants’ (Cunha Neto, 2023). These complex stem structures likely enhance and optimise stem integrity against mechanical disturbances, favouring flexibility and toughness over stiffness and brittleness (Caballé, 1998; Rowe *et al*., 2004; Ménard *et al*., 2013).

Vascular variants are found in lianas with diverse stem shapes (e.g. circular, oval square, lobed and ribbon‐shaped), often integrating different configurations of wood and secondary phloem produced by a single or multiple bifacial vascular cambia. These variants result in a remarkable diversity of structures, combining lignified tissues such as xylem and/or fibres with softer tissues such as secondary phloem and/or unlignified parenchyma (Schenck, 1893; Philipson *et al*., 1971; Caballé, 1998; Rowe *et al*., 2004, 2006; Angyalossy *et al*., 2012, 2015; Trueba *et al*., 2015; Chery *et al*., 2020b). These complex structures are believed to resist mechanical disturbances by enhancing flexibility and toughness, thereby preventing catastrophic failure of mechanical integrity and maintaining hydraulic conductivity in long, slender stems (Fisher & Ewers, 1991; Ménard *et al*., 2013). Observations and experiments have indeed shown that the alternation of soft parenchyma and stiffer mechanical tissues reduces stiffness (lower elastic modulus) and enhances compliance, flexibility and toughness (e.g. Fisher & Ewers, 1991; Speck & Rowe, 1999). The tissue heterogeneity creates ‘fracture‐stopping’ properties, which allow energy at the tip of a developing crack or fracture to dissipate as it encounters softer tissue patches within the wood cylinder (Cook & Gordon, 1964; Vincent, 1990; Vogel, 2013; Alexander, 2018). This mechanism likely protects vital tissue functions by promoting benign stem splitting and separation of the stem rather than catastrophic cross‐sectional rupture. It also limits structural damage from tension, twisting, or bending, and facilitates repair after injury (Dobbins & Fisher, 1986; Fisher & Ewers, 1991; Putz & Holbrook, 1991; Angyalossy *et al*., 2012). Overall, both elastic mechanical properties and resistance to catastrophic failure appear to depend largely on the degree of homogeneity and compartmentalization of stiff and compliant tissues (Philipson *et al*., 1971; Dobbins & Fisher, 1986; Fisher & Ewers, 1991; Putz & Holbrook, 1991; Ménard *et al*., 2013). Therefore, the ability of lianas to endure significant stem deformation without losing functionality depends on their complex, heterogeneous stem anatomy (Putz & Holbrook, 1991). Considering this anatomical diversity within a community is thus crucial to better understand species coexistence and strategies, as well as the distribution and survival of lianas in different and changing environments.

The phylogenetic diversity of vascular variants has been extensively described since the 19^th^ century, beginning with the work of Schenck (1892) and since then strengthened by others such as Obaton (1960), Carlquist (1991), Pace *et al*. (2009) and more recently categorized based on developmental and evolutionary characteristics of vascular development by Angyalossy *et al*. (2015), Chery *et al*. (2020b) and Cunha Neto (2023). Studies have focused on vascular variants at various taxonomic levels, including families like Bignoniaceae (Pace *et al*., 2009; Angyalossy *et al*., 2012), or orders like Piperales (Isnard *et al*., 2012; Wagner *et al*., 2014; Trueba *et al*., 2015), and many more (e.g. Wagner *et al*., 2012; Chery *et al*., 2020a). Functional and biomechanical analyses of lianas have explored how mechanical properties such as stiffness and flexibility evolve from young to older stages across different phylogenetic groups and vascular variants (Putz & Mooney, 1991; Rowe & Speck, 1996; Rowe *et al*., 2006). Mechanical and anatomical studies have also been used in evolutionary contexts to investigate how tissues have adapted over time, as well as how mechanical properties change with ontogeny and respond to ecological factors (Gallenmüller *et al*., 2001, 2004; Lahaye *et al*., 2005; Isnard *et al*., 2012; Wagner *et al*., 2012, 2014; Isnard & Feild, 2015; Trueba *et al*., 2015).

Overall, the diversity of vascular variants seems to play a crucial role in shaping key functional traits, such as resistance and resilience to damage, repair mechanisms, and hydraulic conductivity – considered essential for liana survival. While these aspects have been explored at the individual level in the early 1990s (Fisher & Ewers, 1991; Putz & Holbrook, 1991), their implications at the community level remain poorly understood. Recent advances have revealed high phylogenetic and functional diversity in liana stems, with convergent anatomical plans underlying adult stem mechanics and hydraulics. This may explain their remarkable adaptive success in disturbed and changing environments (Angyalossy *et al*., 2015; Rowe & Speck, 2015). However, surprisingly few studies have explored how anatomical diversity translates into functional roles at the community level, raising several questions: (i) how structural diversity varies within a given community, (ii) whether this diversity involves functional and ecological trade‐offs, and (iii) how different stem structures are linked, or not, with different ecological strategies.

Except for several pioneering efforts to quantitatively assess anatomical diversity within a community (e.g. Caballé, 1986a in Gabon; Yang *et al*., 2022 in Taiwan), anatomical studies at the community level remain scarce. Although these studies did identify anatomical variations within specific communities, they did not address how the putative functional diversity relates to anatomical diversity. Community‐level research is essential to understanding how anatomical combinations and associated functions – such as stiffness, flexibility, water conduction, damage repair, and storage – shape species' functional preferences, survival strategies, and cohabitation dynamics.

In this paper, we explore how vascular variants and wood traits contribute to understanding the functional life strategies of co‐existing lianas in a site in Northern Congo. In this context, we define liana functional anatomical traits as structural features of liana anatomy – such as vessel arrangements, wood density, or vascular variants – that influence key physiological and mechanical functions of lianas. Water transport efficiency, mechanical flexibility, and resistance to mechanical stress are all crucial for liana survival in dynamic forest environments. Studying these traits and their interactions is key for understanding the life history strategies of lianas, and especially how they balance investment in structural support, hydraulic function, and resilience in competitive forest environments. The main goal of this study is to incorporate underexplored, complex functional traits – such as juvenile wood and vascular variants – into the framework of liana ecological strategies. This approach aims to provide a deeper understanding of their life histories and bridge the gap between species‐level and community‐level studies. Accordingly, we address the following questions:

1. What is the anatomical diversity of vascular variants in liana species of Northern Congo and what are the possible approaches of upscaling and understanding this diversity at the community level?
2. Do functional anatomical traits highlight different liana life history strategies at the community level?
3. Are different vascular variants associated with different ecological strategies?

To achieve this, we collected liana stem samples from areas near permanent scientific plots and categorized functional types of vascular variants based on wood geometry, component tissues, and the degree of vascular tissue division and compartmentalization. The simplification of categories (by necessity) allowed us to investigate the diversity of vascular variants and address the challenges of studying complex structures in community‐scale studies. We further identified anatomical traits linked to self‐supporting and climbing (non‐self‐supporting) phases in the liana growth habit. By integrating these ‘new’ liana traits with classic wood traits, we explored liana life‐history strategies within the community and their links with the diversity of forms exhibited by lianas.

## Materials and Methods

### Study site and liana stem collection

Our study was conducted in a semi‐deciduous forest in the north of the Republic of Congo, in the Loundoungou forest management unit of the logging company Olam‐Agri (2°27′11.87″N and 17°02′32.17″E). The experimental site was created in 2014 for monitoring trees over space and time under different silvicultural treatments and was established in an abiotically homogeneous area in terms of soil and topography (Freycon, 2014; Kaçamak *et al*., 2022, 2025). It is composed of four 9‐ha plots, located on a plateau with elevations between 395 and 470 m above sea level with sandy‐clay to clay‐sandy soils, where all trees ≥ 10 cm in diameter have been measured annually since 2015 (Freycon, 2014; Kaçamak *et al*., 2025). From April to July 2017, we launched liana monitoring inventories in regularly spaced 144 quadrats of 20 × 20 m^2^ within the permanent plots, where all lianas ≥ 1 cm in diameter at 1.3 m were identified as vernacular taxa and their diameters measured. More information about ground inventories and the permanent plots can be found in Kaçamak *et al*., 2025.

Destructive sampling, necessary for anatomical study, was conducted in areas adjacent to permanent plots to avoid disrupting long‐term monitoring, while still representing the liana communities within the plots. Field collection occurred in September to October 2021 along transects radiating from the edge of a 20‐m buffer around the permanent plots to a maximum distance of 1 km, considering the surroundings as an equivalent environment to the permanent plots because of the homogeneous abiotic nature of the study area (Kaçamak *et al*., 2025). Eight samples of mature lianas were, however, taken between 8 and 20 m from the permanent plot margins because they were not found outside the buffer, after checking that the stems were not growing towards and establishing within the permanent plots. We sampled stems of at least 2 cm in diameter at 1.3 m from their basal rooting point on the ground, following established international protocols (Gerwing *et al*., 2006; Schnitzer *et al*., 2008), on climbing individuals (no juvenile self‐supporting stems). Detailed functional and particularly anatomical studies using paraffin embedding and sectioning are time consuming and cannot be carried out with high levels of replication. Moreover, they might depend on very specific local effects impacting the individual rather than reflecting broadly comparable effects over a large number of samples and replicates (Rowe & Speck, 2015). We ensured as far as possible that sampled climbing adult liana stems had similar diameters at 1.3 m length from their rooting point to allow comparisons at the same or similar ontogenetic developmental stage. To account for the potentially high variability of individual tree‐liana situations and the wide range of possible ontogenetic stages of attached lianas in the field, we nevertheless collected the following additional pieces of information for each individual.

For each liana stem collected, we recorded the exact location (Garmin gps), identified the species whenever possible, measured the diameter at 1.3 m from their rooting point, and noted the following observations: bark description, climbing mechanism (twining, hook attachment etc. when observable), approximate height of first attachment and stage of climbing development – whether the liana was still climbing or had already reached the canopy. We used a sharp hand saw to cut a segment of stem *c*. 10 cm long, measuring the stem diameter in the middle of the excised segment, which was temporarily stored moist in plastic bags. They were then stored by submerging in 40° alcohol in plastic bottles during transport to the laboratory before processing and taking high quality images (as described in the section below).

In total, we collected 164 stem segment samples with diameters between 2.0 and 6.4 cm (median 3.2 cm), belonging to 67 mature liana species, with an average of 2.5 individuals per species or morphospecies, hereafter referred to as species (minimum of 1 sample and maximum of 6 samples per species). This sampling covered, at the community level, 45 taxa, representing 95% of all liana individuals monitored in the permanent plots in the 2017 liana inventory (5926 individuals out of a total of 6206; Supporting Information Table S1).

### Liana stem processing and trait acquisitions

In the laboratory, each stem sample was divided into two parts. The first segment was used to measure liana wood density (described in ‘Anatomical functional traits and measurements’ in the Materials and Methods section). The second segment was first polished on one side with a water‐assisted, rotary grinder using a decreasing series of sandpaper grades with 8 different grain sizes, from FEPA P60 (260 μm grain sizes) to FEPA P1200 (14 μm grain sizes). The entire cross‐section was then scanned with an Epson Stylux Perfection V700 scanner at a resolution of 2400 ppp.

#### Functional categorization of vascular variants

The goal and a key issue were to categorize liana stems in the simplest way possible by: using classic criteria that represent the diversity of tissue organizations described in previous pioneering studies mentioned above in the introduction (e.g. Schenck, 1892; Obaton, 1960; Carlquist, 1991; Angyalossy *et al*., 2015; Chery *et al*., 2020b; Cunha Neto, 2023); using newly formulated traits, particularly with regard to the degree of heterogeneity of tissue types known to reflect stiffness and toughness characteristics of the stem (e.g. Philipson *et al*., 1971; Dobbins & Fisher, 1986; Fisher & Ewers, 1991; Putz & Holbrook, 1991; Speck & Rowe, 1999; Ménard *et al*., 2013); and macroanatomical characteristics that are discernible on images, from which it was possible to reproduce categorisations on large samples at the community level.

Faced with a large diversity of anatomical patterns resulting from highly variable growth of the bifacial vascular cambium, we elected to provisionally group macroanatomical organizations using the high‐resolution scans according to: the cross‐sectional geometry of the secondary xylem; and the degree of subdivision of the vascular tissue produced by the bifacial vascular cambium. We distinguished liana stems varying from simple, complete, uncompartmentalized wood cylinders to more complex forms, with different degrees of compartmentalization showing, for example, marked transverse alternations of stiff and soft tissue types (Rowe & Speck, 2005; Isnard & Silk, 2009; Ménard *et al*., 2013).

**Fig. 1:**
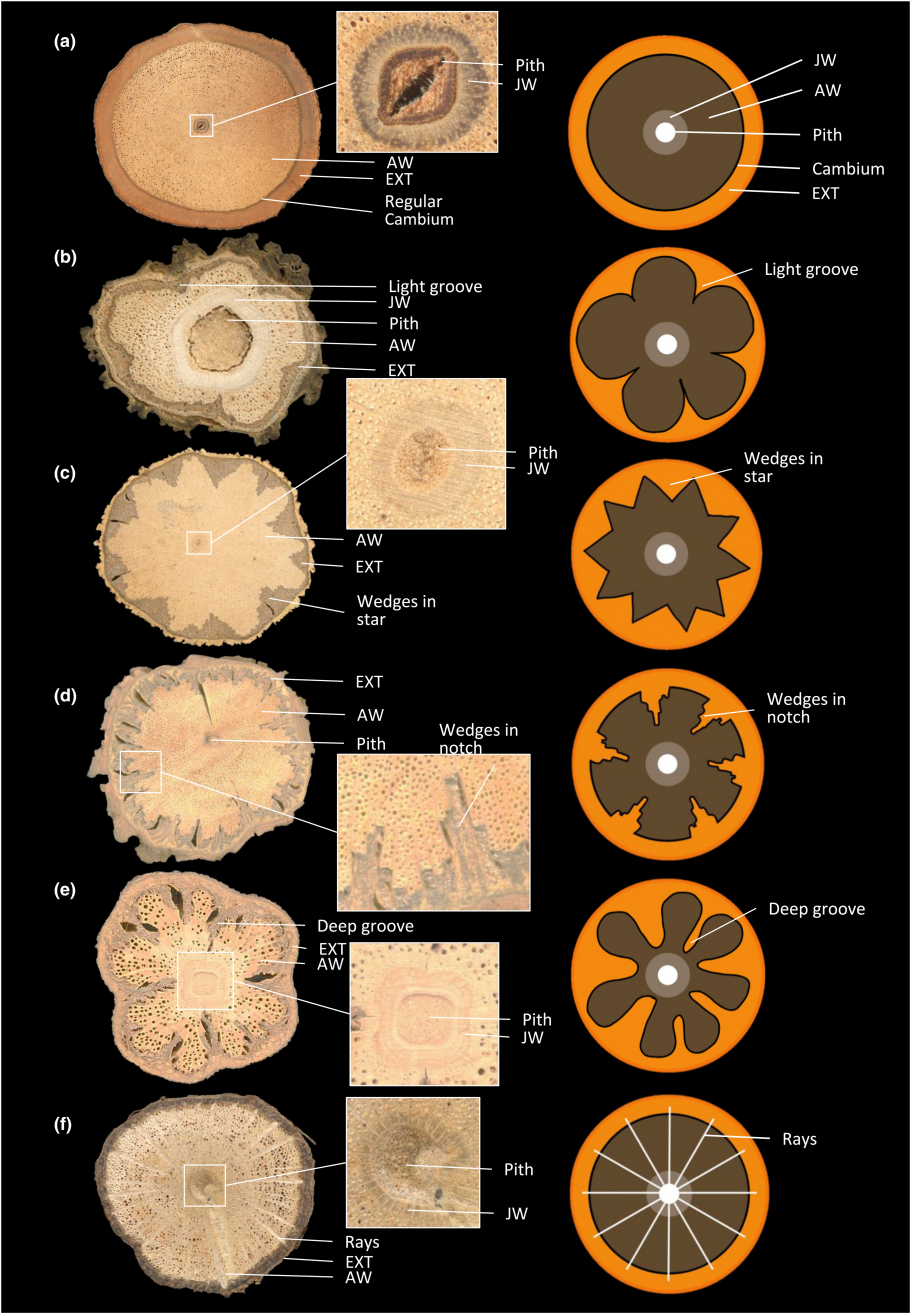
Uniform wood and vascular variant types. (a) *Landolphia landolphioides* (Hallier f.) A.Chev. (Apocynaceae), regular secondary growth; (b) *Manniophyton fulvum* Müll.Arg. (Euphorbiaceae), furrowed xylem with shallow phloem wedges; (c) *Loeseneriella* sp.2 (Celastraceae) and (d) *Strophanthus preussii* Engl. & Pax (Apocynaceae), furrowed xylem with phloem wedges from atypical cambial activity; (e) *Loeseneriella* sp.1 (Celastraceae), furrowed xylem with phloem wedges from atypical cambial activity; (f) *Tetracera podotricha* Gilg (Dilleniaceae), axial vascular elements in segments. AW, adult wood; EXT, external phloem, cortex, bark and all other nonconductive tissues; JW, juvenile wood.

**Fig. 2:**
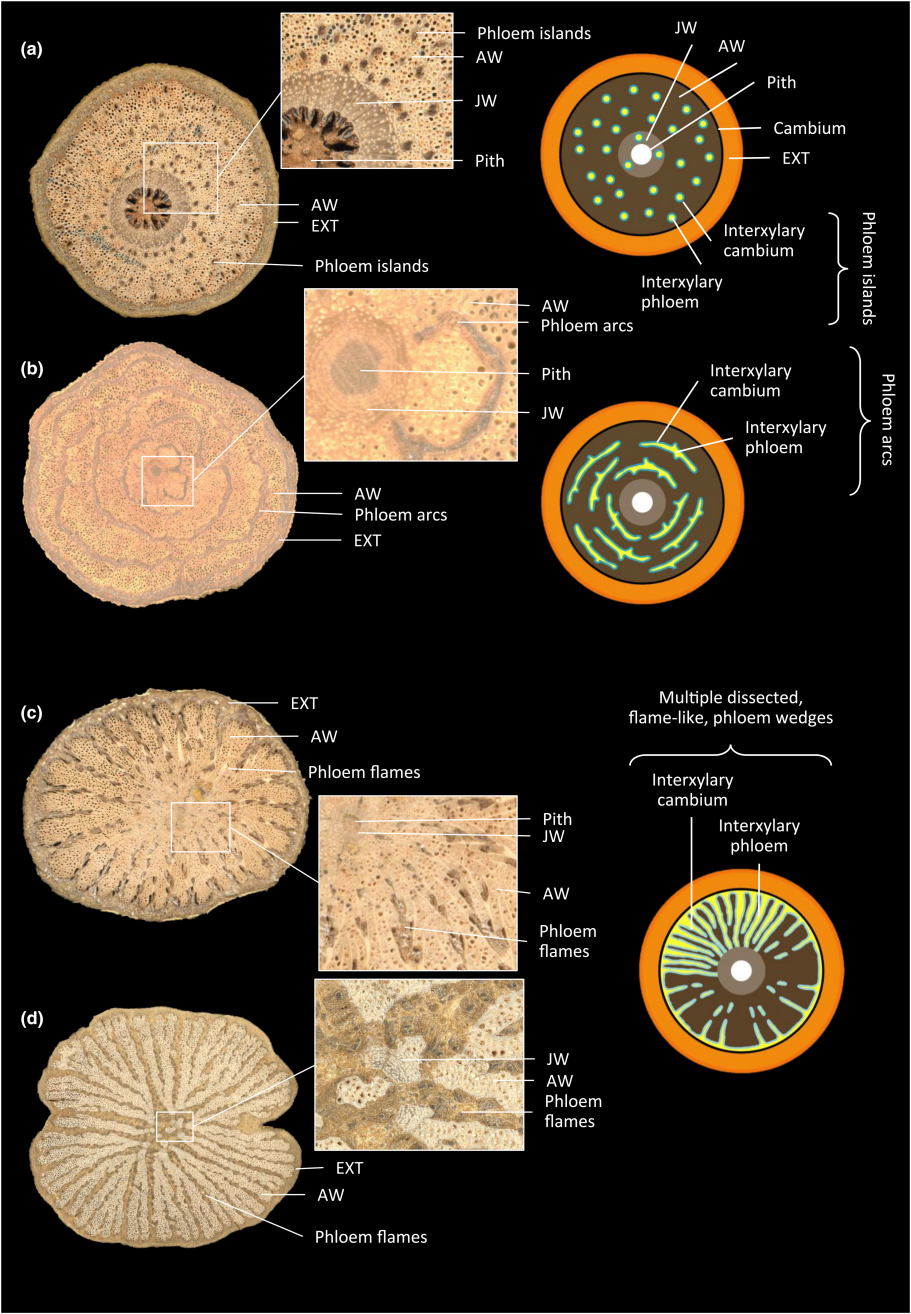
Internal phloem, wood types and vascular variant types. (a) *Strychnos* sp.1 (Loganicaceae) and (b) *Celastraceae* spp. (Celastraceae), interxylary phloem with cambial inclusions; (c) *Loeseneriella* sp.2 (Celastraceae) and (d) *Mokasso* (vernacular sp.), multiple dissected phloem wedges. AW, adult wood; JW, juvenile wood; EXT, external phloem, cortex, bark and all other non‐conductive tissues.

**Fig. 3:**
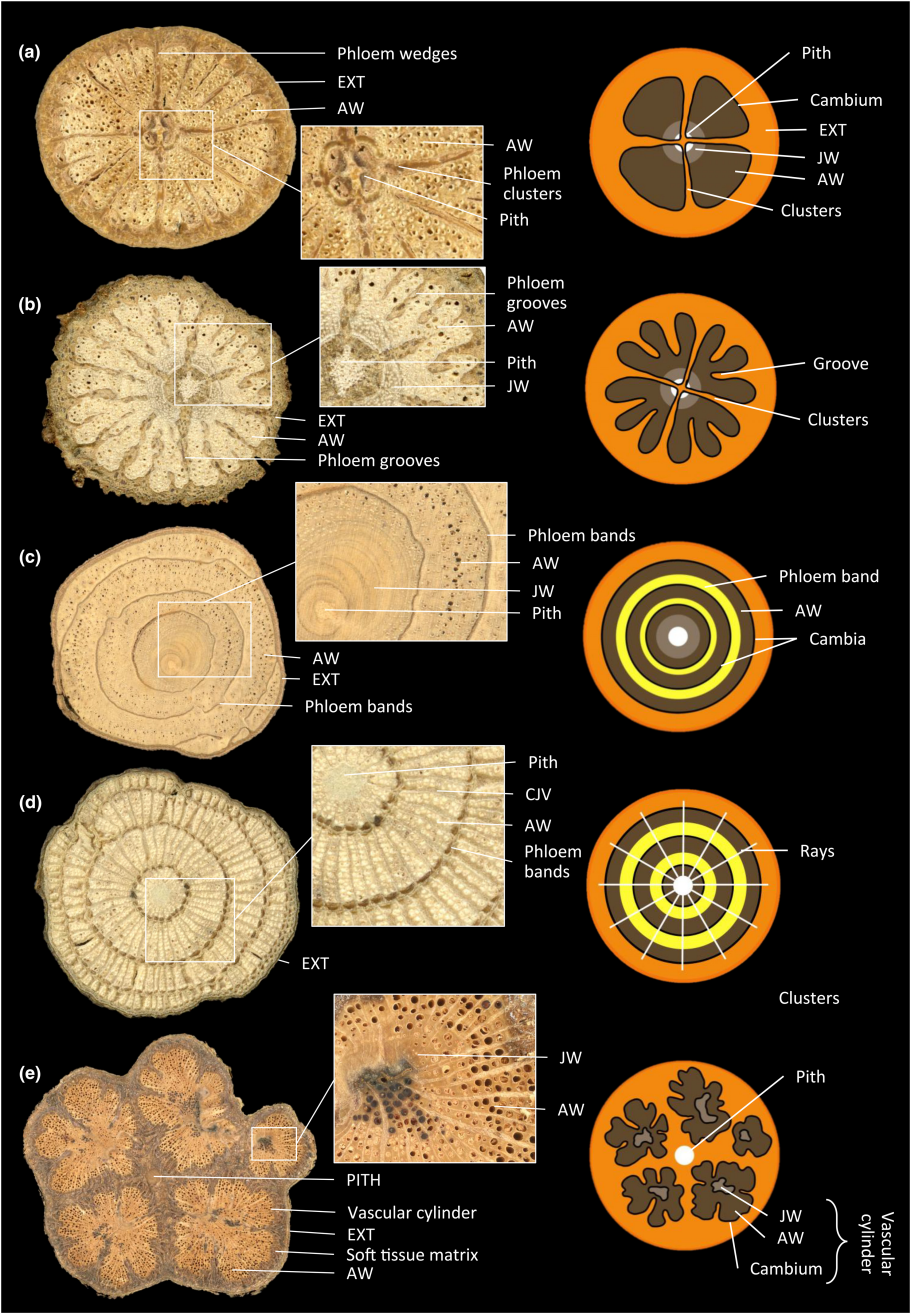
Compound wood and vascular variant types. (a) *Neuropeltis velutina* Hallier f. (Convolvulaceae) and (b) *Turraea vogelii* Hook.f. (Meliaceae), dissected xylem; (c) *Agelaea pentagyna* (Lam.) Baill. (Connaraceae) and (d) *Triclisia macrophylla* Oliv. (Menispermaceae), successive cambia; (e) *Loeseneriella* sp.1 (Celastraceae), compound. AW, adult wood; JW, juvenile wood; EXT, external phloem, cortex, bark and all other non‐conductive tissues.

We distinguished three categories of wood structure. (1) Uniform woods, where the wood cylinder is undivided and entire, with different degrees of lobing of the outline of the cylinder. (2) Internal phloem woods, where the main component is still the entire and complete wood cylinder but which has different types of inclusions of soft tissues. (3) Compound woods, where the wood cylinder has divided developmentally into independent parts from one vascular cylinder or has developmentally formed multiple vascular cylinders. These categories were further differentiated into 10 different vascular variants (vascular variant types), shown in Figs 1, 2, 3, to explore how more detailed features might enhance the information for inferring functional strategies. These vascular variants were differentiated based on the different anatomical patterns and tissue arrangements following the biomechanical studies, and not on the evolutionary origins of the patterns. Therefore, some species may fall into different wood structure categories depending on their life history stage, as usually anatomical patterns of liana stems complexify with time. This high throughput anatomical approach provided a simplified yet broad and inclusive way to delineate liana stem organisation and likely functionality for ecological analysis. The different categories and each vascular variant within each category are described in Notes S1. Additional information about anatomical configurations is provided in Table S2 for liana samples which differed from typical vascular variants. After establishing different categorizations for the stems that were present at the site, we observed for all individuals of the same species how vascular variants varied from an ecological and developmental point of view.

#### Anatomical functional traits and measurements

We selected eight functional anatomical stem traits to explore liana ecological trends. These are related to different life‐history functions, including early‐life history strategies of lianas – the self‐supporting phase – their resilience to disturbance and perturbation, and their hydraulic safety. While anatomical compartmentalization plays an important role in the mechanical and hydraulic properties of adult liana stems faced with environmental perturbation, the juvenile wood takes up a different role in the early life of lianas, providing young self‐supporting liana stems with mechanical stiffness (Fisher & Ewers, 1991; Rowe *et al*., 2004; Rowe & Speck, 2005). We explored the potential roles of juvenile wood as well as the development and anatomical structure of vascular variants in relation to different life‐history strategies and additional more classical anatomical traits (see plant traits studied in Table 1).

We performed liana wood density measurements using the Archimedes thrust technique as described by Birouste *et al*. (2014). All samples were first debarked (removing the bark and external phloem layer) and saturated with water under negative pressure for 48 h, through several vacuum‐negative pressure cycles (−1 to 0 bar). We then measured the Archimedes thrust with the water displacement method. Debarked stems were then oven dried at 40°C for 10 d, to avoid damaging water‐saturated tissues at extreme temperatures, and then at 103°C for 48 h to eliminate all water. Finally, we measured the mass of the dried stem samples to assess liana wood density. It must be pointed out, however, that the wood density measurements for lianas do not measure only wood tissue. Even after debarking, most mature liana stems contain a mixture of wood as well as significant amounts of softer non‐woody tissues, such as parenchyma and interxylary phloem (through vascular variants). This contrasts with many other growth forms such as trees, which have a much more homogeneous cylinder of wood, and thus where the presence of non‐woody tissues is far less.

**Table 1:** Anatomical functional traits, their definitions and their range for 136 liana individuals.

Life history strategies	Anatomical functional traits	Abbreviation	Range	Unit	Functional role
Early‐life	Pith area	Pith	[0–1.96]	mm^2^	Rigor of searcher stems & mechanical spacer^1^
	Juvenile wood area	JW	[0–2.33]	mm^2^	Cost of self‐supporting phase, investment into stiff initial stem structure^2^
	Adult, juvenile wood and interxylary phloem area	Mix_Wood	[0–0.34]	mm^2^	Increase volume and speed of photosynthate transport^3^
Resilience to disturbance	External tissues (external phloem, cortex and bark area)	Ext	[0–13.49]	mm^2^	Protection of vital tissues and defense against pathogens^4^
	Heterogeneity index	Hetero_index	[0.09–2.14]	No unit	Stress dissipation and fracture point stopping^5–7^
	Phloem : Xylem ratio	ratio_PX	[0–1.08]	No unit	Elasticity and stiffness of wood and fracture point stopping^6,9^
Hydraulic safety	Wood density	WD	[0.18–0.70]	g cm^−3^	Mechanical structure, transport, longevity and defense against pathogens^10^
	Wood vessel density	vessel_density	[1.76–30.37]	No. mm^−2^	Conductance efficiency^10–12^
	Wood vessel area	mean_vessel	[0.004–0.177]	mm^2^	Conductance safety^12,13^

^1^Olson *et al*. (2018); ^2^Rowe *et al*. (2004); ^3^Carlquist (2013); ^4^Paine *et al*. (2010); ^5^Putz & Holbrook (1991); ^6^Fisher & Ewers (1991); ^7^Dobbins & Fisher (1986); ^8^Caballé (1998); ^9^Ménard *et al*. (2013); ^10^Poorter *et al*. (2010); ^11^Sperry *et al*. (2008); ^12^Aloni & Zimmermann (1983); ^13^Sperry *et al*. (2006).

**Fig. 4:**
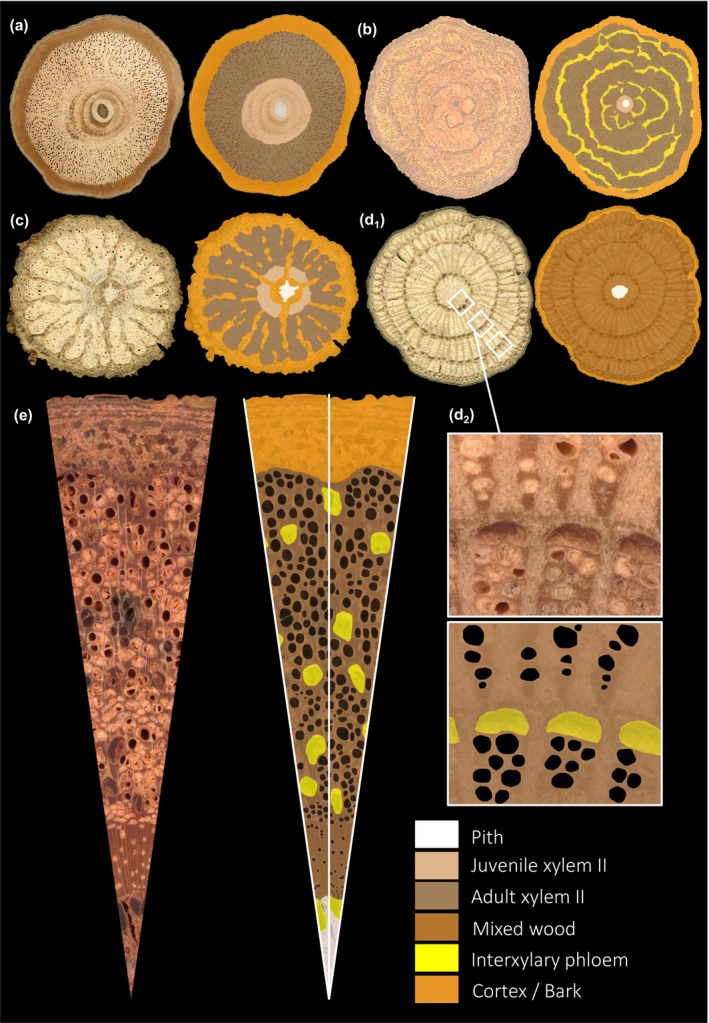
Original images of the stem cross‐sections with the corresponding anatomical tissues and vessel delineations. (a) *Landolphia robustior* (K.Schum.) J.G.M.Pers. (Apocynaceae) with a regular secondary growth. (b) *Celastraceae* spp. (Celastraceae) with interxylary phloem with cambial inclusions as phloem arcs. (c) *Turraea vogelii* (Meliaceae) with dissected xylem. (d) *Triclisia macrophylla* (Menispermaceae) with successive cambia organised with axial elements and conjunctive tissue. (e) *Strychnos* sp.1 (Loganiaceae) with interxylary phloem with cambial inclusions as scattered phloem islands. [Correction added on 27 August 2025, after first online publication: the right‐hand image in (e) has been corrected.]

All other traits were measured through manual delineations of tissue areas on high‐resolution images using scans of the entire cross‐sections or through finer resolution images of a radial segment on a microscope, in Photoshop and numerically analyzed using ImageJ software (Fig. 4). For entire cross‐sections, we measured the areas of the pith, juvenile secondary xylem (juvenile wood), adult secondary xylem (adult wood), interxylary phloem, and all external tissues (including the external phloem and all other non‐conductive tissues such as parenchyma, cortex and bark), as presented in Fig. (4a–d_1_).

For fine‐scale measurements, we took high‐resolution images of a radial segment depending on the size and intactness of each segment, which was centered at the centroid of the pith and extended to include the outermost perimeter of the stem. For a given stem section, we selected the largest radial segment – if the stem diameter was variable, as is often the case. Variable radial widths for a given liana stem can have a significant influence on cross‐sectional shape, but also the complexity of the tissues produced by the vascular variant. Thus, the protocol ensured that we measured the ‘maximum’ development for a given stem section. Anatomical details of the surfaces of the radial segments were captured using a Keyence VHX 7000 microscope with resolutions between 4850 and 19 600 ppp. We delineated and measured all vessel areas (entire vessels; Fig. 4e, black‐coloured areas) and the entire wood area. More details about the challenges of vessel delineations are stated in Notes S2. We also delineated the interxylary phloem area in samples for which it was not clearly visible on scans (Fig. 4e, yellow). From these measurements, we derived vessel density, mean vessel diameter, and phloem : xylem surface ratio. We also measured a value representing the macroscopic tissue heterogeneity of each sample. This comprised the mean number of tissue types traversed by three radial lines extending from stem centre to outer stem surface, that is along the two lateral edges and the middle of each radial segment (Fig. 4e, white lines). This value was normalized to each radial line length to consider different stem diameters and size factors. The mixed wood area (Mix_Wood in Table 1), including the juvenile and adult wood, and when present, the areas of interxylary phloem, was also standardized by the diameter of each sample.

One type of liana organization comprised a striking organization of radially arranged bands (successive cambia), including pockets and bundles of secondary xylem and phloem vascular tissue (known as ‘caps’), often with tangentially alternating bands of wide‐ray tissue (Fig. 4d_2_). Because of the highly intricate organization of such forms and the time that would have been required for manual vessel delineation, we measured the mean vessel, wood, and phloem cap areas for three sub‐sections (Mennega, 1982). These measurements were made along a radial transect of the vascular cylinder cross‐section at the inner, middle, and outer positions of stems showing a successive cambial organisation with axial elements and conjunctive tissue (Fig. 4d_1_). The mean vessel density, diameter, and phloem : xylem ratio were computed as the mean values of the three sub‐sections.

### Statistical analyses

To explore how anatomical traits were associated with each other and with vascular variants, we carried out a principal component analysis (PCA) with all quantitative traits. We described the main trends and their variations by wood structure category and vascular variant types. To assess if wood structure categories and vascular variants differed significantly along the first two PCA axes, we carried out Wilcoxon pairwise tests, with or without a Bonferroni correction. To control for the effect of phylogenetic relatedness among species on trait correlations, we adopted a second analysis. We first built a phylogenetic tree using DNA extracted from liana leaf samples collected in May 2018 at the study site, targeting two plastid regions, rbcl and matk (see Kaçamak *et al*., 2025 for details). We constructed a maximum likelihood phylogenetic tree using the best fitted nucleotide substitution model for calculating pairwise distances (GTR + G + I), implemented in the MEGA software (Kumar *et al*., 2018). We then used this tree to fit a phylogenetic generalized linear mixed model (PGLMM) to all functional traits independently (Ives *et al*., 2020). This model includes species as a random effect, with phylogenetic covariance specified by the phylogenetic tree. We then extracted the residuals from the PGLMM models for each trait, representing the variation in traits after accounting for phylogenetic effects. These residual traits were used to perform a second PCA to compare trait associations with and without the effects of phylogeny. We then computed pairwise log–log linear models for a subset of traits to explore in detail some trait relationships. The first PCA and log–log linear models were carried out on samples where all anatomical functional traits were measured, for a total of 136 samples belonging to 60 liana species; overly damaged samples were excluded. The second PCA analysis controlling for the effect of both species' identity and evolutionary history (phylogenetic relatedness) was carried out on a subset of 44 species (117 individuals) for which we had phylogenetic information.

**Table 2:** Wood structure categories and vascular variants of the collected 164 liana stem samples.

Uniform wood	Regular secondary growth	Furrowed xylem with shallow phloem wedges	Furrowed xylem with phloem wedges from atypical cambial activity	Furrowed xylem with deep phloem wedges	Axial vascular elements in segments	Total
	79	12	4	5	20	120
Internal phloem wood	Interxylary phloem with cambial inclusions		Multiple dissected phloem wedges	–	–	
	20		4	–	–	24
Compound wood	Dissected xylem		Successive cambia	Compound		
	4		13	3		20

All statistical analyses were made with the statistical program R, v.4.2.1 (R Core Team, 2022). We used the packages ade4 for multivariate analysis (Dray & Dufour, 2007), phyr for phylogenetic analysis (Ives *et al*., 2020), ape for the creation of a phylogenetic tree (Paradis & Schliep, 2019) and ggplot2 for visualisations (Villanueva *et al*., 2016).

## Results

### Taxonomic and vascular variant diversity of lianas from northern Congo

Among the 164 samples collected, 73% had uniform wood and 27% had complex wood forms (15% with internal phloem wood and 12% with compound wood; Table 2). Most samples had a regular secondary growth (48%; Fig. 1a), followed by axial vascular elements in segments (12%; Fig. 1f) and interxylary phloem with cambial inclusions (12%; Fig. 2a,b), successive cambia (8%; Fig. 1b,c) and furrowed xylem with shallow phloem wedges (7%; Fig. 3d).

**Fig. 5:**
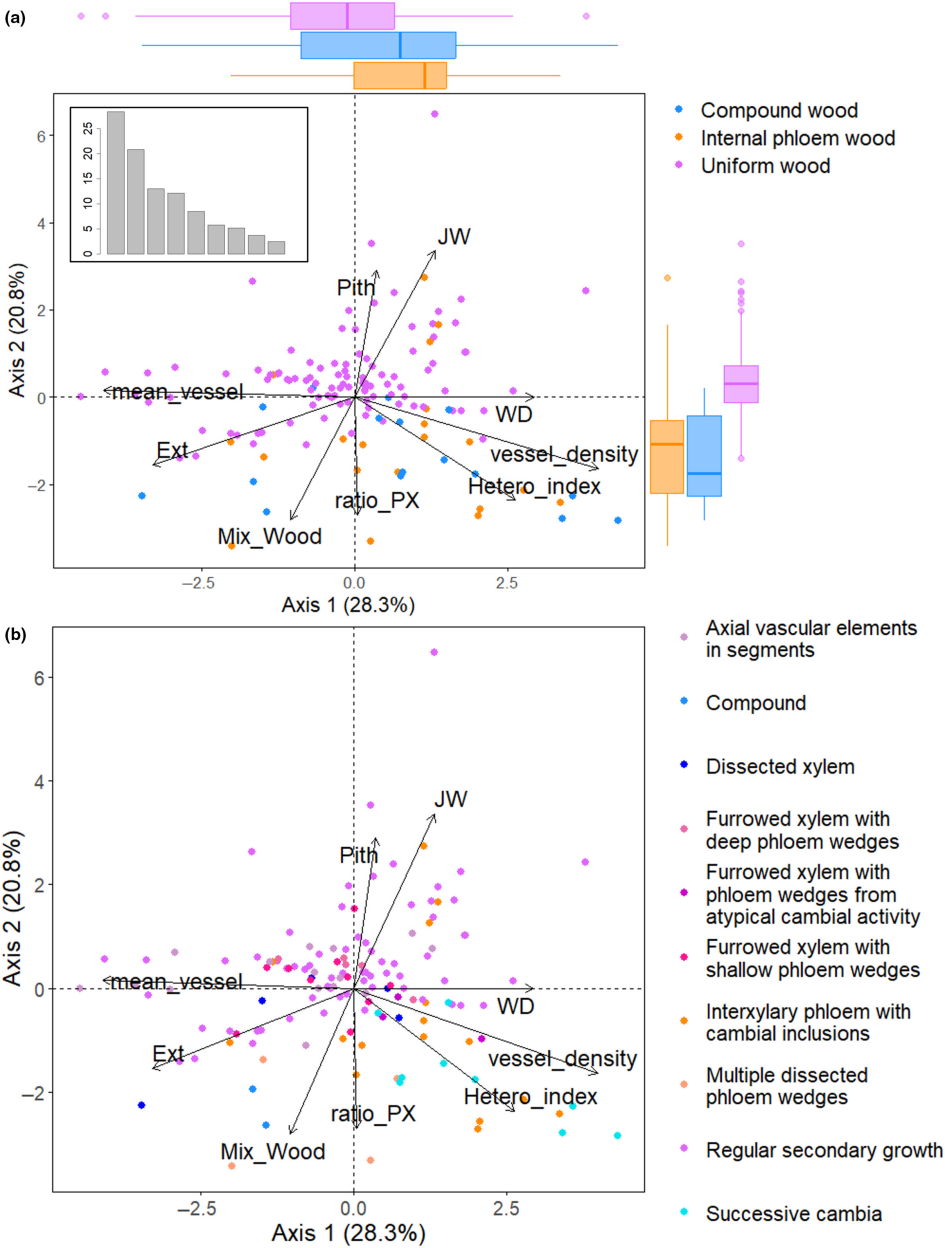
Principal Component Analysis performed on anatomical functional traits. (a) Wood structure categories; uniform, internal phloem and compound woods; are represented in pink, orange, and blue, respectively. Boxplots illustrate the differences between the scores of wood structure categories along the two first PCA axes. Eigenvalues (%) are represented in the top left corner. (b) Vascular variant types are represented in different colours. Ext, external tissues; Hetero_index, heterogeneity index; JW, uvenile wood area; mean_vessel, mean wood vessel area; Mix_Wood, adult, juvenile and interxylary phloem area; Pith, pith area; ratio_PX, phloem : xylem ratio; vessel density, wood vessel density; WD, wood density.

**Fig. 6:**
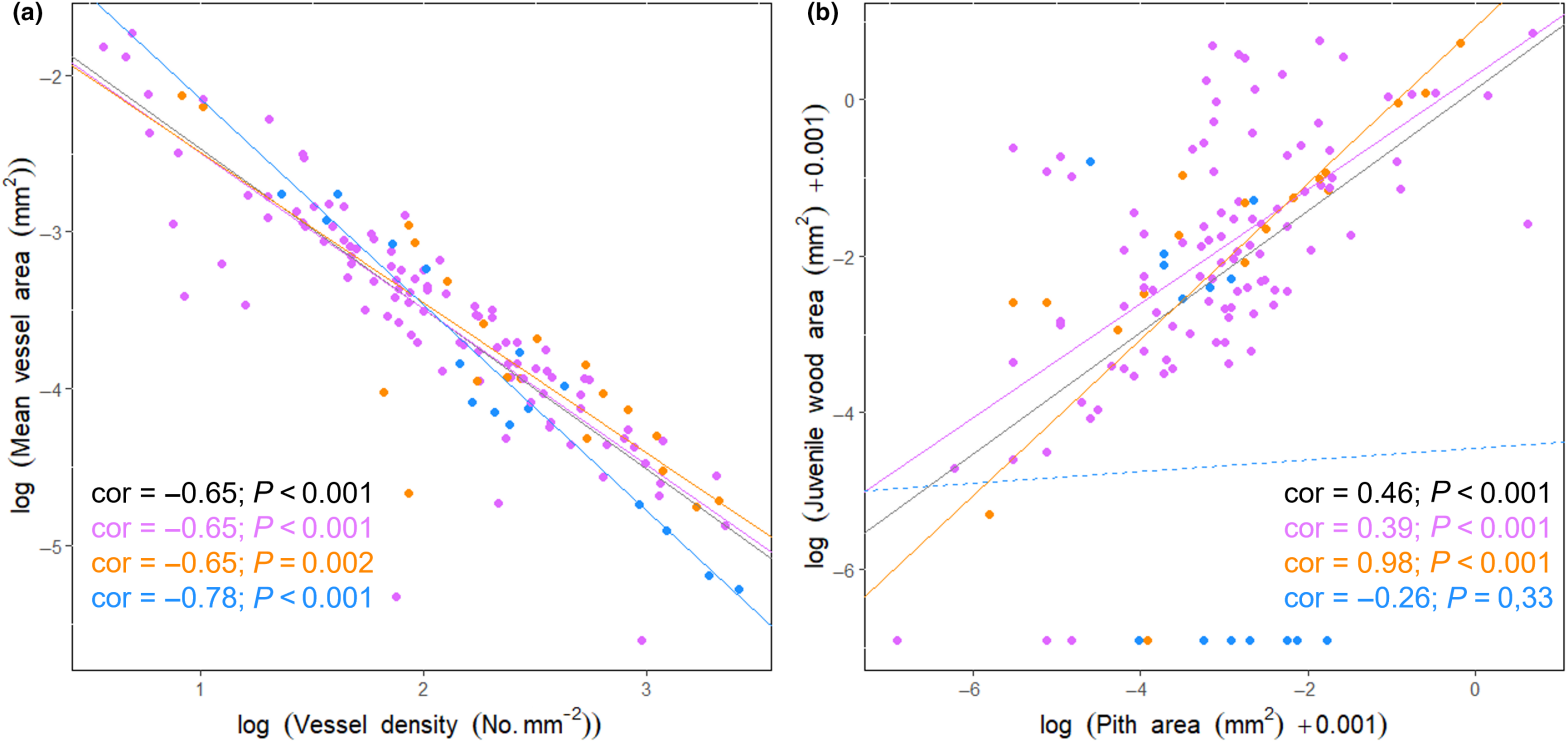
Log–log relationship between (a) the vessel density and the mean vessel area, (b) the pith and juvenile wood area. The Pearson correlations (cor) and associated *P*‐value (*P*) are displayed. The lines illustrate the predicted linear log–log model by wood structure categories, with dashed lines when it is not significant and with full lines when the relationship is significant on each graph. Wood structure categories – uniform, internal phloem and compound woods – are represented in pink, orange and blue, respectively. The grey line represents the model including all categories.

**Fig. 7:**
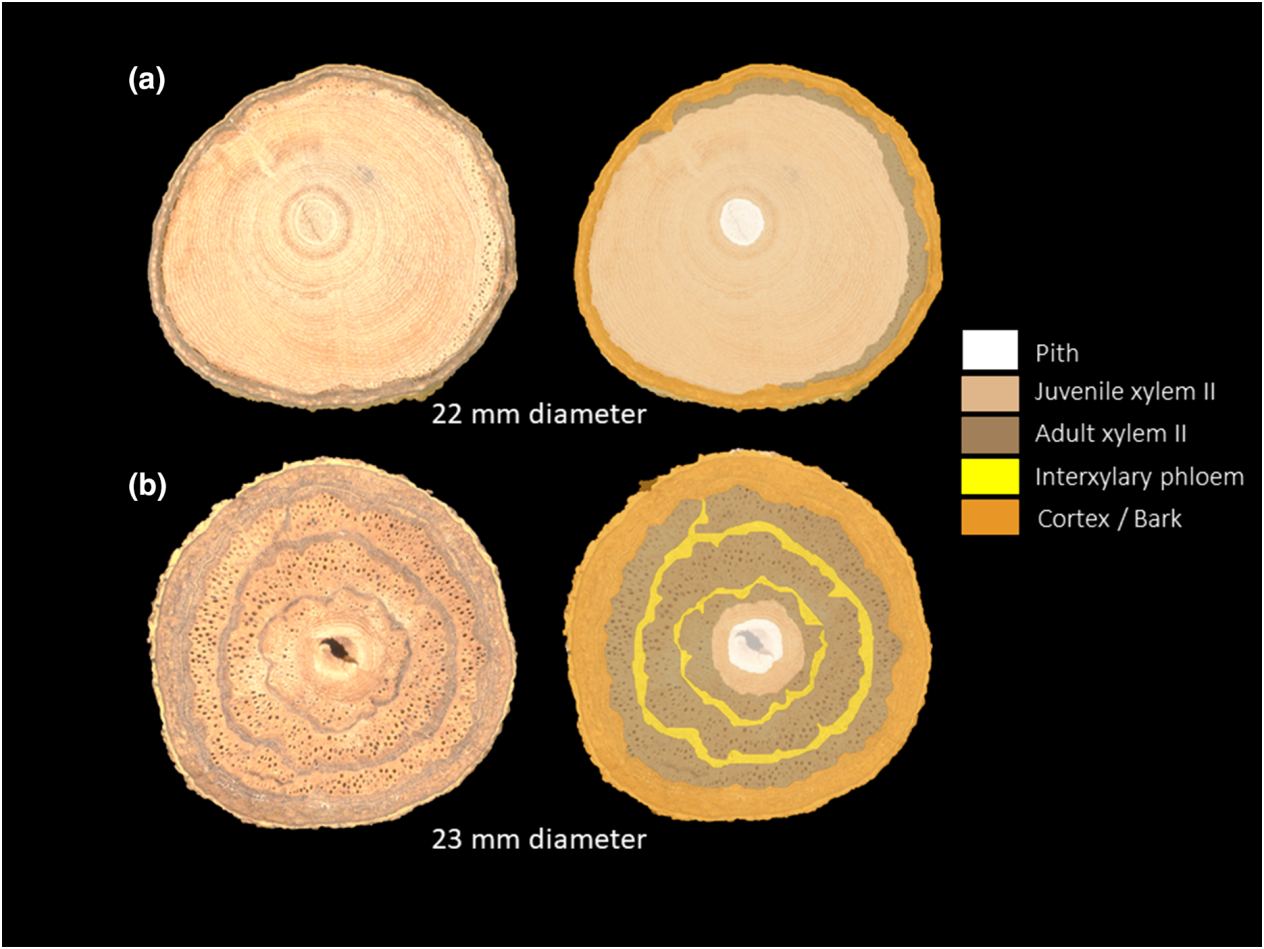
Different vascular variant development in stems of the same species, *Salacia* sp.2 (Celastraceae), with similar diameters but different compartmentalization levels. (a) Shows a large development of juvenile wood, while (b) a small extent of juvenile wood but expresses a complex vascular variant (interxylary phloem with cambial inclusions).

Out of the total 67 species, for 41 species we collected more than one sample, allowing us to explore variations of taxon‐specific vascular variants. Within those 41 species, 90% of them exhibited taxon‐specific anatomical wood configurations (e.g. uniform, internal‐phloem or compound woods), while only 10% of liana species (four species) presented variable anatomical configurations. These four species all belonged to the Celastraceae family, within the genus *Salacia* or *Loeseneriella* (Celastraceae, Table 3), and appeared to be capable of exhibiting simple configurations (regular secondary growth or furrowed xylem with phloem wedges) as well as very complex ones (included interxylary phloem with cambial inclusions or compound or dissected xylem). Additionally, some species appeared to alternate between degrees of variable lobing and the outline of the wood cylinder (e.g. regular secondary growth, furrowed xylem with shallow phloem wedges and axial vascular elements in segments) for simple wood configurations. We observed 12 species in which individuals showed different vascular variants within the same wood structure category (all uniform woods) and within the diameter range applied in the sampling (Table 3).

**Table 3:** Different vascular variant types and wood structure categories within species.

Family	Species scientific names	Wood structure category	Vascular variant types	Variant types observed ratio
Connaraceae	*Agelaea* sp.3	Uniform wood	Regular secondary growth/Furrowed xylem with shallow phloem wedges	3 : 2
Apocynaceae	*Baissea multiflora*	Uniform wood	Regular secondary growth/Furrowed xylem with shallow phloem wedges	2 : 1
Apocynaceae	*Baissea subrufa*	Uniform wood	Regular secondary growth/Furrowed xylem with shallow phloem wedges	4 : 1
Connaraceae	*Cnestis corniculata*	Uniform wood	Regular secondary growth/Furrowed xylem with shallow phloem wedges	2 : 1
Euphorbiaceae	*Manniophyton fulvum*	Uniform wood	Regular secondary growth/Furrowed xylem with shallow phloem wedges	2 : 4
Apocynaceae	*Strophanthus preussi*	Uniform wood	Regular secondary growth/Furrowed xylem with phloem wedges from atypical cambial activity	1 : 2
Lamiaceae	*Clerodendrum* sp.2	Uniform wood	Regular secondary growth/Axial vascular elements in segments	1 : 1
Combretaceae	*Combretaceae* spp.	Uniform wood	Regular secondary growth/Axial vascular elements in segments	1 : 1
Passifloraceae	*Passifloraceae* spp.	Uniform wood	Regular secondary growth/Axial vascular elements in segments	1 : 1
Dilleniaceae	*Tetracera podotricha*	Uniform wood	Regular secondary growth/Axial vascular elements in segments	1 : 2
Fabaceae	*Dalhousiea africana*	Uniform wood	Furrowed xylem with shallow phloem wedges/Axial vascular elements in segments	2 : 3
Fabaceae	*Leucomphalos brachycarpus*	Uniform wood	Regular secondary growth/Furrowed xylem with deep phloem wedges/Axial vascular elements in segments	2 : 1 : 2
Celastraceae	*Salacia* sp.1	Uniform wood/Internal phloem/Compound wood	Furrowed xylem with deep phloem wedges/Multiple dissected phloem wedges/Dissected xylem	1 : 1 : 1
Celastraceae	*Loeseneriella* sp.1	Uniform wood/Compound wood	Furrowed xylem with deep phloem wedges/Furrowed xylem with phloem wedges from atypical cambial activity/Compound	2 : 1 : 1
Celastraceae	*Loeseneriella* sp.2	Uniform wood/Internal phloem	Furrowed xylem with phloem wedges from atypical cambial activity/Interxylary phloem with cambial inclusions	2 : 2
Celastraceae	*Salacia* sp.2	Uniform wood/Internal phloem	Regular secondary growth/Interxylary phloem with cambial inclusions	1 : 1

Overall, we found that the majority of liana species exhibited simple anatomical configurations (uniform woods), while complex configurations (internal phloem and compound woods) were fewer but diverse. Most species seemed to exhibit taxon‐specific wood configurations (e.g. uniform, internal‐phloem or compound woods) but seemed labile in the degree of lobing of the outline of the wood cylinder.

Within the plots, the proportions of taxa with wood structure types were similar to those we collected from the adjacent area, with a majority of simple configurations with 85.5% of uniform wood lianas, while 14.5% presented complex anatomical structures (8% with internal phloem wood and 6.5% with compound wood). The five most abundant vascular variants within the liana community were taxa with furrowed xylem with shallow phloem wedges (53.2% of all individuals), followed by axial vascular elements in segments (16.4% of all individuals), regular secondary growth (13.9% of all individuals), interxylary phloem with cambial inclusions (8% of all individuals), and successive cambia (3.8% of all individuals) types. Given the comparable relative proportions of anatomical configurations between our samples from the liana plot community and the adjacent area, the analyses of their anatomical strategies below (‘Multivariate and bivariate anatomical trait associations’ in the Results section) can thus be representative of the diversity of ecological strategies of lianas within our community.

### Multivariate and bivariate anatomical trait associations

PCA performed on functional anatomical traits presented two dominant axes, representing 49.1% of the variation, corresponding to two putative ecological strategies of lianas, on which all variables were well represented (Fig. 5). While the first axis seemed more related to the association between hydraulic traits, the second axis seemed to associate traits in relation to the early‐life and resilience characteristics of lianas. After accounting for phylogenetic relatedness, we found that trait correlations and dominant axes on the residuals PCA remained consistent with the general PCA (Fig. S1).

The first strategy axis contrasted lianas with a low density of large vessels, low wood densities, and thick external tissues against lianas with a high density of small vessels, high wood densities, and thin external tissues. The second axis opposed lianas with simple anatomical organisations with large piths and large juvenile wood cylinders against lianas with complex anatomical organisations (often with developmental successions of different tissue types) characterized by small piths and little to no juvenile wood.

Anatomical configurations of lianas (e.g. uniform, internal phloem or compound woods; Fig. 5a) mostly varied along the second PCA axis with anatomical traits. Indeed, on the first axis, we found that lianas with different wood structures exhibited significant differences only between uniform woods and internal phloem woods (Wilcoxon's *W* = 650, *P* = 0.014; Bonferroni‐adjusted *P* = 0.041). However, along the second axis, lianas with simple anatomical organisations were significantly different from all complex anatomical organisations respectively, between uniform wood lianas and internal phloem woods (*W* = 1614, *P* < 0.0001; Bonferroni‐adjusted *P* < 0.0001) and compound woods (*W* = 1466, *P* < 0.0001; Bonferroni‐adjusted *P* < 0.0001).

The first two PCA axes did not segregate well the 10 vascular variant types with an apparent large overlap of trait values among these types (Fig. 5b). Nevertheless, we found significant differences of PCA scores between vascular variants on both PCA axes (Kruskal Wallis, chi‐squared = 32.6, *P* < 0.001 for axis 1 and chi‐squared = 49.9, *P* < 0.0001 for axis 2), suggesting that some vascular variants might have distinct strategies. For example, species with successive cambia (turquoise in Figs 5b, 4d_1_) have a higher density of small vessels, higher wood densities, and were also characterized by small piths and little or no juvenile wood. This was confirmed by pairwise Wilcoxon tests where only successive cambia differed significantly on the first PCA axis from more simple vascular variants such as regular secondary growth (*W* = 11, *P* < 0.001; Bonferroni‐adjusted *P* = 0.028), furrowed xylem with shallow phloem wedges (*W* = 1, *P* < 0.001; Bonferroni‐adjusted *P* = 0.017) and axial vascular elements in segments (*W* = 78; *P* < 0.001; Bonferroni‐adjusted *P* = 0.017). Along the second PCA axis, successive cambia also differed significantly from the same three simpler vascular variants. Moreover, other complex forms, such as interxylary phloem with cambial inclusions and multiple dissected phloem wedges, both internal phloem wood structures, also differed significantly from the simple regular secondary growth forms along the second PCA axis (*W* = 244, *P* < 0.001, Bonferroni‐adjusted *P* = 0.041; and *W* = 1, *P* < 0.001, Bonferroni‐adjusted *P* = 0.043; respectively).

Among functional traits shaping the first strategy axis, we found a strong linear relationship between mean vessel area and vessel density, the heterogeneity index, and external tissue area (Fig. 6). The strongest relationship was a negative one between liana vessel density and mean vessel area (cor = −0.65; *P* < 0.001; Fig. 6a), with increasingly smaller vessels observed as vessel density increased, a tendency observed for all wood structure configurations. Nevertheless, simpler stem organisations (low heterogeneity index) tended to have larger vessels, while more complex and heterogeneous anatomical configurations tended to favour smaller vessels on average (cor = −0.27, *P* = 0.001). These lianas with simpler forms also tended to display thicker external tissues surrounding their vascular cylinder as vessel size increased (cor = 0.37, *P* < 0.001). Liana stem wood density was also significantly associated with liana hydraulic traits. Lianas with lower wood densities exhibited significantly lower vessel densities (cor = 0.36, *P* < 0.001) and wider vessels (cor = −0.5, *P* < 0.001).

Interestingly, there were no significant relationships between liana wood density and the heterogeneity index of liana anatomical configurations, nor the interxylary phloem ratio. Liana wood density variations were similar among anatomical configurations, regardless of whether they had a uniform internal phloem or compound wood structures (Fig. S2).

Among the functional traits shaping the second strategy axis, we found the strongest positive relationship between juvenile wood and pith area (cor = 0.46; *P* < 0.001; Fig. 6b). Lianas with larger piths tended to produce thicker juvenile wood cylinders. While this tendency seemed to be the case for simple uniform and internal phloem wood lianas, it did not consistently apply to more complex lianas with compound woods. Lianas with compound wood configurations tended to develop either a little or no juvenile wood, while uniform wood lianas tended to produce a noticeable amount of juvenile wood.

## Discussion

In this study, we collected stem samples of mature, climbing co‐existing liana taxa to explore the functional anatomical diversity of the liana community and their putative strategies. We identified two primary functional ecological strategy axes associated with liana stem characteristics. The first axis was mainly related to hydraulic traits and water‐use strategies, with relationships with the heterogeneity index of anatomical configurations, the thickness of external tissues, and wood densities. The second axis mainly distinguished liana species through their early‐life (pith and juvenile wood traits) and resilience strategies. Significant differences in wood anatomical configurations aligned particularly with this strategy axis, distinguishing simple configurations (uniform wood) from more complex ones (internal phloem and compound woods). While taxon‐specific anatomical configurations were prevalent (only 10% of species showed variable wood configurations), we found some plasticity in vascular variants within liana species, raising further challenges and implications that we discuss below.

### Liana anatomical ecological strategies

#### Hydraulic safety vs efficiency trade‐off and potential water‐use adjustment strategies

The first axis of the PCA exploring stem anatomical trait associations included the trade‐off between the number and size of vessels, from liana species with a low density of large vessels to those with a high density of small vessels. As vessel and lumen sizes increase, water flow and hydraulic conductivity increase exponentially (Aloni & Zimmermann, 1983; Ewers, 1991; Sperry *et al*., 2006). However, wider vessels are generally considered more susceptible to failure due to embolism (Ewers *et al*., 1990), although some studies dispute this, as no clear mechanistic explanation has been demonstrated in the tropics (Lens *et al*., 2022; Isasa *et al*., 2023). Overall, the relationship between mean vessel diameter and vulnerability to embolism remains debated, with multiple interacting factors likely at play, such as pit membrane thickness, conduit connectivity, or the presence of vasicentric tracheids (Levionnois *et al*., 2021; Isasa *et al*., 2023; Olson *et al*., 2023). Altogether, this trade‐off between hydraulic conductivity, safety and efficiency is well documented for tree species (Baas, 1986; Sperry *et al*., 2008; Poorter *et al*., 2010), and is important for a better understanding of their functioning, performance, and distribution in the environment (van der Sande *et al*., 2019; Zhang *et al*., 2023). Compared to tree vessel dimensions, lianas have consistently been found to have wider vessels, presumably to compensate for their slender stem form (Ewers *et al*., 1990; Zhu & Cao, 2009), regardless of the vegetation type (Zhang *et al*., 2023). This pattern has been shown for co‐occurring lianas and trees (Zhu & Cao, 2009) but has been less documented among liana species (shown in a seasonally dry forest by De Guzman *et al*., 2016). On the other hand, some studies have found a decoupled relationship between liana hydraulic efficiency and safety (van der Sande *et al*., 2019; Zhang *et al*., 2019), although lianas appear to have comparable variability in vessel size to that of trees (Meunier *et al*., 2020). While controlling for stem length, liana vessel diameters have been found to be indistinguishable from those of self‐supporting plants (Rosell & Olson, 2014). However, lianas seem to exhibit a wider range of vessel diameters, with a significantly higher abundance of narrow vessels (see below for the discussion on vessel dimorphism (Carlquist, 1985a; Olson *et al*., 2023)).

Plants have evolved several strategies to efficiently transport water upward while avoiding embolisms (Zhang *et al*., 2023). They are known to adjust vessel structure and organisation by changing the size and density of the vessels; this has not always been shown to correlate with all lianas (van der Sande *et al*., 2019; Zhang *et al*., 2019). Alternatively, lianas are well known for adjusting vascular configurations. One documented adjustment is xylem dimorphism, through vessel dimorphism (Carlquist, 1981) where small narrow vessels adjacent to the wider vessels within the stem cross‐section can create hydraulic bridges to prevent embolism (Angyalossy *et al*., 2015), or vasicentric tracheids, where tracheids serve as an auxiliary conducting system that supplies water to leaves when nearby vessels fail due to air embolism (Carlquist, 1985b, 1988), as found in oak trees (Wheeler & Thomas, 1981). These structures are regarded as an evolutionary strategy (as is all dimorphism) and as a survival mechanism to adapt to extreme environments (Carlquist, 2014; Olson *et al*., 2023). Vessel dimorphism can thus likely incorporate safety mechanisms which allows lianas to maintain high hydraulic conductivity, but also provide safety measures against embolism and avoiding cavitation under extreme conditions (Carlquist, 1985b, 2014; van der Sande *et al*., 2019; Zhang *et al*., 2023).

Another highly visible but much less‐documented adaptation towards stem hydraulic safety includes the diversity of vascular variants. Although lianas that coexist with trees have wider vessels (Zhu & Cao, 2009; Zhang *et al*., 2023), the hydraulic conductivity is less affected by twining and twisting than the more homogeneous organization of typical trees, and they are also able to maintain water flow in surviving, unbroken wood connections between two fractured stem segments (Putz & Holbrook, 1991). This ability to resist mechanical constraints without losing entirely stem mechanical function and hydraulic conductivity is directly related to stem anatomy. Interestingly, in this study, we found that only lianas with internal phloem wood (and not compound woods) had significantly different water‐use strategies than lianas with uniform wood structures. This suggests that lianas with internal phloem wood tend to favour hydraulic conductivity safety over efficiency. Interestingly, lianas in the internal phloem wood category are characterized by pockets of soft tissue within the wood cylinder, generally consisting of phloem but also including parenchyma. Recent studies suggest that stem parenchyma in lianas may play a role in hydraulic conductivity, facilitating xylem water transport by contributing to the repair and refilling of obstructed or embolized vessels (Secchi *et al*., 2017; Kawai *et al*., 2022; Zhang *et al*., 2024). Additionally, interxylary phloem has been found to favour the conduction of photosynthates, whereas the more typical external phloem specializes in storage (Pace *et al*., 2011; Angyalossy *et al*., 2012; Carlquist, 2013). We also found thicker external tissues in lianas having wider vessels. The role of these outer tissues, from the external phloem to the cortex and the bark, is primarily to protect from external damage, such as from xylophagous organisms, and may also protect the inner part of the stem from other kinds of mechanical damage and fracture propagation (Paine *et al*., 2010; Poorter *et al*., 2014). Vascular variants may play a role in helping lianas maintain high hydraulic efficiency while still being safe against embolism through the inclusion of soft tissues in the wood cylinder, favouring conductance, or external tissues to protect vital functions. Nevertheless, we found little evidence that vascular variants and wood structure categories might influence the parameters of the relationship between vessel width and density. Indeed, there did not appear to be any major differentiation of the vascular variants in terms of vessel size and density. Vascular variants did not seem to be associated with specific water‐use strategies, but they may still underlie alternative strategies for hydraulic efficiency and safety. However, finer‐scale mechanisms are difficult to assess at the level of investigation employed in our study. Further research is needed to better understand how water‐use strategies are combined with vascular variants.

Overall, lianas appear to exhibit diverse water‐use strategies through different adaptations aimed at maintaining both high hydraulic efficiency and safety. These adaptations may influence, for example, the distribution of liana species along hydrological gradients, providing safety mechanisms to ‘drought vulnerable’ species in valleys, or conferring growth advantages to ‘drought resistant’ species on plateaux with limited access to the water table (Oliveira *et al*., 2019; Rocha *et al*., 2022). Further studies are needed to investigate how these adaptations shape liana species distribution across different topographical environments.

#### Early‐life strategies and potential associated resilience

The second axis of the PCA opposed traits related to early‐life and resilience strategies of lianas. It distinguished lianas with simple forms (uniform wood structure lianas), characterized by large piths and juvenile wood, from more complex forms. Though pith is usually a parenchymatous tissue with little mechanical stiffness, it has been found in some studies to facilitate stem rigidity in terms of bending and torsion by enhancing the second moment of area of mechanically stiff tissues (fibres and dense wood) which are placed outside the pith and thus further from the neutral axis (in bending) of the plant stem. The pith thus acts as a geometrical spacer for physiologically costly dense stiff tissues and thus increases stem rigidity over stems with smaller piths (Mahley *et al*., 2018; Olson *et al*., 2018), thus providing relatively ‘cheap’ mechanical stability. This feature has recently been shown to be a commonly deployed mechanical trait enhancing the reach of liana searchers (the maximum distance of self‐supporting stems before attaching to a host or losing their self‐supporting habit; Hattermann *et al*., 2022). Lianas with larger piths tended to have searchers with longer reaches. Juvenile wood has also been found to increase the maximum length of searchers before finding a mechanical host to climb on (Ewers & Fisher, 1991) including higher proportions of juvenile wood when there is a scarcity of hosts (Rocha *et al*., 2022). We found that larger areas of pith and juvenile wood were contrasted against high heterogeneity and mixed wood proportions associated with decreasing stiffness, likely providing elasticity to allow twisting and bending (Dobbins & Fisher, 1986; Rowe *et al*., 2004; Ménard *et al*., 2009; Angyalossy *et al*., 2015). Those lianas, which are composed of complex vascular variants – internal phloem wood and a compound wood structure – are potentially less vulnerable to disturbance, with a high potential ability of recovery after injury (Fisher & Ewers, 1989, 1991).

Overall, lianas appear to exhibit two different early‐life strategies that may be related to resilience strategies as they mature. They either have a lengthier self‐supporting phase with a well‐developed early‐life strategy with more efficiency to search hosts with longer and stiffer searching stems. This would tend towards thriving in environments where there is a scarcity of host supports or allow them to cross large gaps between widely spaced branches and canopies. Alternatively, other stem types seem to show no particular early‐life strategy but tend to favour flexibility through heterogeneous tissue configurations, and are thus potentially less vulnerable to disturbances, and might thus be more adapted to very dynamic forests. Further studies are needed at this point to explore whether the longer expression of juvenile characteristics (Lahaye *et al*., 2005) or the preference for resilience characteristics might explain species distribution and success in disturbed environments. Overall, analysing functional trade‐offs at the broader wood category level (unique, internal‐phloem or compound woods) appeared to provide more insights at the community level than specific vascular variant types.

#### Revisiting the notion of resilience for lianas

Exploring the potential functional patterns through associations of liana wood density and other anatomical traits, we found that liana wood density was mainly related to water‐use strategies. Consistent with other studies, liana wood density was significantly related to water flow (Ewers *et al*., 1990; Meunier *et al*., 2020; De Guzman *et al*., 2021). We found that lighter‐wooded liana species had significantly lower vessel densities and wider vessels. However, this negative relationship between wood density and hydraulic efficiency (van der Sande *et al*., 2019) was not systematic for all liana species, where, for example, Zhang *et al*. (2023) found one species with extremely wide vessels but not light wood. On the other hand, lianas with a high density of small vessels, supposedly favouring hydraulic safety, exhibited higher wood densities, while some studies have found no relationship between wood density and stem vulnerability to cavitation (De Guzman *et al*., 2021). Interestingly, liana wood density was also not associated with any traits related to the resilience of lianas, including the different anatomical configurations, the tissue heterogeneity index, nor the interxylary phloem ratio. The use of wood density as a trait for indicating resilience, as it is frequently used for trees (Putz *et al*., 1983; Van Gelder *et al*., 2006; Poorter *et al*., 2010), is probably not appropriate for lianas – at least old stages of flexible growth. Mature and adult non‐self‐supporting lianas, which rely for survival primarily on the combination of stem flexibility, toughness (resistance to fracture propagation) and high conductance, have very different mechanical properties compared with mature and adult self‐supporting trees that rely on stability and strength (Rowe & Speck, 2005). Lianas and trees have been found to have comparable ranges of wood density values (Putz & Holbrook, 1991; Meunier *et al*., 2020), but they exhibit significant differences in bending and torsion stiffness and rotation‐to‐break characteristics (Putz & Holbrook, 1991). The ability of lianas to resist disturbances and to survive depends on their complex and heterogeneous stem anatomy, which favours flexibility over stiffness and toughness over strength (Putz & Holbrook, 1991; Caballé, 1998; Rowe *et al*., 2004). One study has even showed increased brittleness in domesticated manioc, which are lianas whose mature stems have less dense wood and do not compartmentalize as wild ones do (Ménard *et al*., 2013). So, wood density should be used with caution when studying lianas and interpreting resilience, as it can reflect hydraulic efficiency, but because of the profound difference in life‐history mechanics, may not be fully related to survival and resilience as it is purported for trees.

However, vascular variants may serve as a mechanical strategy for the liana to adapt and survive in unstable environments (Rowe & Speck, 2005). In the field, for example, we observed that damaged *Loeseneriella* sp.1 exhibited a compound stem form after separating into several separate strands at the site of injury and subsequently reformed as a single stem (personal observation; Fig. S3), which was also observed by Caballé (1986b) on *Loeseneriella* and demonstrated by Fisher & Ewers (1991) with experimentally induced injuries. Few studies, such as Fisher & Ewers (1991), have reported the different repair mechanisms and damage shown by vascular variants. Further studies linking vascular variants and strength and survival, and following the pioneering studies of Putz & Holbrook, Fisher & Ewers and Dobbins, are crucially needed to explore the biomechanical properties associated with anatomical configurations and functional traits related to resilience to validate the tendencies that we observe in this study.

### Anatomical diversity, ontogenetic and environmental plasticity and their importance in trait studies

Most species in our study exhibited taxon‐specific anatomical configurations. However, we still observed some variation at the level of wood structure (e.g. uniform, internal phloem or compound woods), and especially at the level of vascular variants of lianas with simple and uniform wood structure, with different degrees of lobing of the outline of the wood cylinder. Vascular variants can be taxon‐specific, and some families are known to display a specific vascular variant (Caballé, 1986a). For example in the Menispermaceae, all species appear to have a compound wood with successive cambia and wide rays with conjunctive tissue (*Tiliacora* sp.1 and *Triclisia macrophylla* in our case; Mennega, 1982; Carlquist, 1996; Ortiz *et al*., 2007; Angyalossy *et al*., 2012).

Nevertheless, a large plasticity in development exists between individuals of the same species or genus. This variability between anatomical configurations may result from ontogenetic but also environmental plasticity. During their ontogeny, liana stems tend to gain in complexity as they grow in size, as we observed for the Celastraceae species, *Loeseneriella* sp.1 (Fig. S4). In general, the cambium appears to maintain a potential for ontogenetic plasticity and in particular growth form variability during evolution, particularly among lignophytes – plants with a bifacial vascular cambium, since the Devonian period (Speck & Rowe, 2003). Indeed, in angiosperms, the first study to reconstruct the evolution of stem ontogeny in the genus *Paullinia* revealed that nearly all vascular variant forms evolved from a common ancestral form (Chery *et al*., 2020b), suggesting a degree of plasticity and freedom in vascular variant types. Variability of anatomical configuration may also be due to the surrounding environment. We observed two individuals of the same species, *Salacia* sp.2, with similar diameters (respectively of 22 and 23 mm) with completely different anatomical development (Fig. 7). One exhibited only juvenile wood, whereas the other displayed a little juvenile wood but also developed a vascular variant having interxylary phloem with cambial inclusions (internal phloem wood structure). Despite this difference in organisation, both individuals had already reached the canopy. While the existence of a juvenile phase seems to be ontogenetically similar to all lianas (Caballé, 1998), the timing of its expression is likely dependent on the spatial contingencies and time required to find support (Rowe & Speck, 2005) in the surrounding environment (Soffiatti *et al*., 2022; Lima *et al*., 2024).

Thus, the values of stem traits measured for a liana stem largely depend on the extent of its juvenile phase (Rowe & Speck, 2015). Transitioning from juvenile to adult results in changes in wood density, vascular characteristics, and tissue configurations to adapt to the new mechanical demands of the lianescent habit (Fisher & Ewers, 1991; Rowe & Speck, 2005). Most liana species exit their juvenile stage and start producing a lianescent (adult) xylem instead of a non‐lianescent (juvenile) xylem when they attach to a host and begin climbing (Soffiatti *et al*., 2022; Lima *et al*., 2024). The timing of this expression, that is, the extent of the juvenile phase, very likely depends on local environmental variables at the community level. On a broader scale, the juvenile to lianoid transition underlies a wide potential for complex plasticity and intraspecific variation of stem traits across diverse liana species. Furthermore, as this study has shown, the juvenile‐adult switch can be followed by marked variations in the onset of later lianoid ontogenetic stages (Obaton, 1960; Putz & Holbrook, 1991; Rowe & Speck, 2005; Nesheim & Økland, 2007). Finally, not all liana species tend to exhibit or even develop a juvenile, self‐supporting phase, as observed in this study, suggesting that profoundly different degrees of plasticity exist between liana species (Rowe & Speck, 2015).

In our study area, we found that 86.2% of the liana community showed development of vascular variants, but only 14.5% showed compartmentalized forms with internal phloem or compound wood structures. By contrast, a rather different proportion was observed by Caballé in a northeastern forest in Gabon where 42% of lianas showed internal phloem or compound wood structures (Caballé, 1986a). One very real factor influencing efforts to ‘characterize’ representative vascular variants is the role that the environment plays in changes made to stem development making it even more difficult to determine the originally distinct vascular variant and the life history of lianas. The different local dynamics and past disturbance histories of each site might have a substantial impact on the wood structures exhibited by liana species, reflecting their adaptive response to environmental constraints. Nonetheless, this ‘historical’ aspect requires further research to be fully explored.

Overall, our results indicate that when mature liana stems are collected at the community scale in natural settings, they will potentially include highly variable responses involved in resisting mechanical and hydraulic stress and repair mechanisms to stem damage. This suggests that any interpretation of macro‐anatomical organisation at the community level must consider: the evolutionary diversity of vascular variants as well as the interspecific variation of measured traits; and the fact that both ontogeny and environment significantly influence the expression of vascular variants and functional traits. The combination of all these factors potentially influences the values of all anatomy‐based trait measurements on liana stems. We believe studies such as ours, integrating these difficult aspects of lianas into community level frameworks, are difficult but necessary to explore the intricate complexity of lianas and begin to understand their extraordinary way of life and species distribution over time and space.

## Competing interests

None declared.

## Author contributions

Conceptualization: BK and NR; ground data collection: BK; preprocessing: BK and MG; formal analysis: BK, MR‐M and NR; leaf trait measurements: BK; wood trait measurements: BK and MG; writing (original draft): BK and NR; writing (review and editing): BK, MR‐M, NR, MG, GJLP and J‐JL.

## Disclaimer

The New Phytologist Foundation remains neutral with regard to jurisdictional claims in maps and in any institutional affiliations.

## Supporting information


**Fig. S1** Effect of species identity and phylogenetic structure on trait covariation.
**Fig. S2** Distribution of liana wood density values across liana vascular variants.
**Fig. S3** Stem of *Loeseneriella sp.1* (Celastraceae).
**Fig. S4** Different vascular variant development in stems of species *Loeseneriella* sp.1 (Celastraceae).
**Note S1** Description of wood categories and vascular variants of liana anatomical forms.
**Note S2** Vessel delineations of liana stem samples.
**Table S1** Wood structure category and vascular variants of 45 liana taxa of a Northern Congo liana community.
**Table S2** Wood structure categories and vascular variants of 164 liana samples.
Please note: Wiley is not responsible for the content or functionality of any Supporting Information supplied by the authors. Any queries (other than missing material) should be directed to the *New Phytologist* Central Office.

## Data Availability

Data is available at doi: 10.23708/H2HXEX.
