# Supplementary material for: Linking anatomical diversity with functional life strategies in a liana community of central Africa
Source: New Phytol. 2025 Aug 4;248(3):1225–44. doi: 10.1111/nph.70413 (PMC12489287; doi:10.1111/nph.70413)
Supplement: Supplementary file 1 — Fig. S1 Effect of species identity and phylogenetic structure on trait covariation. Fig. S2 Distribution of liana wood density values across liana vascular variants. Fig. S3 Stem of Loeseneriella sp.1 (Celastraceae). Fig. S4 Different vascular variant development in stems of species Loeseneriella sp.1 (Celastraceae). Note S1 Description of wood categories and vascular variants of liana anatomical forms. Note S2 Vessel delineations of liana stem samples. Table S1 Wood structure category and vascular variants of 45 liana taxa of a Northern Congo liana community. Table S2 Wood structure categories and vascular variants of 164 liana samples. Please note: Wiley is not responsible for the content or functionality of any Supporting Information supplied by the authors. Any queries (other than missing material) should be directed to the New Phytologist Central Office. [file NPH-248-1225-s001.docx]

**New Phytologist Supporting Information**
**Article title:** Linking anatomical diversity with functional life strategies in a liana community of central Africa
**Authors:** Begüm Kaçamak, Maxime Réjou-Méchain, Maël Grolleau, Jean-Joël Loumeto, Grace Jopaul Loubota Panzou, Nick Rowe
**Article acceptance date:** 3 July2025

Table S1. 45 liana taxa representing 95% of all liana individuals of a Northern Congo liana community, their wood structure category and vascular variants.

Note S1. Description of wood categories and vascular variants of liana anatomical forms.

Uniform woods (Figure 1): In this category, secondary xylem (juvenile, inner (depicted in light brown) and adult, outer wood in dark brown) is undivided apart from narrow rays, but the circumference outline of the wood cylinder has different degrees of slight lobing due to variations in cambium activity (black line), creating variations of the outer perimeter. We identified:

- Regular secondary growth (Figure 1, A): Cambial growth is regular (black lines) with homogeneous xylem. The outer perimeter of the wood is circular with a cylinder of secondary xylem (young and adult wood in light and dark brown) surrounded by a cylinder of secondary phloem. The latter is not always macroscopically separable from external cortical tissues and other non-conductive tissues, such as the periderm (orange). Where primary cortex, secondary phloem and periderm cannot be distinguished macroscopically, we refer to this as “bark.” Although this generalization concerning tissues exterior to the wood cylinder is sometimes difficult to apply microscopically, the main focus of identifying different vascular variants based on the vascular tissues still holds.
- Furrowed xylem with shallow phloem wedges (Figure 1, B): Cambial growth is regular but shows different degrees of growth around the perimeter, forming grooves. Lightly uneven cambial growth with differential production of phloem and xylem, where the radial length of the grooves is shorter than half the wood radius.
- Furrowed xylem with phloem wedges from atypical cambial activity (Figure 1, C and D): Cambial growth around the perimeter is more irregular, even ceasing xylem production in some parts of the perimeter, resulting in phloem wedges, furrowing the xylem. The phloem wedges have can have different forms due to the irregular activity of the cambium, such as star-like shapes (Figure 1, C) or rectangular forms (Figure 1, D).
- Furrowed xylem with phloem wedges from atypical cambial activity (Figure 1, E): Highly uneven cambial growth with differential production of phloem and xylem intensifies, creating deep grooves across the wood cylinder, where the radial length of the grooves is greater than half the wood radius.
- Axial vascular elements in segments (Figure 1, F): Axial elements of the vascular cylinder are radially separated by wide rays (greater than one cell wide) of axial parenchyma, sometimes with xylem and phloem tissue, which can extend from the margin of the pith to the outer margin of the secondary phloem. Ray tissues can be lignified or non-lignified (white lines from the pith to the outer phloem in the cortex and bark (orange)).

Internal phloem/parenchyma in wood (Figure 2): In this category secondary xylem is still the main component of the wood cylinder but includes pockets (inclusions) of soft tissue (yellow), generally of phloem and/or parenchyma. Inclusions may appear from the alternate activity of a single cambium, or from the activity of interxylary multiple cambia (light blue). Presence of inclusions leads to 3 different types of vascular variants:

- Interxylary phloem with cambial inclusions (Figure 2, A and B): irregular cambium activity or alternate cambia activity (in light blue) produce phloem arcs that are included within the secondary xylem. This creates macroscopically visible interxylary phloem islands within the wood (yellow; Figure 2, A), or fragmented circles (yellow; Figure 2, B) when there is a higher production of phloem arcs.
- Multiple dissected phloem wedges (Figure 2, C and D): Highly uneven production of phloem and xylem produce deep phloem wedges and a high production of phloem arcs embedded in the xylem cylinder. These confer a “flame-like” appearance to the phloem inclusions.

Compound woods (Figure 3): In this category, secondary xylem is divided into independent parts, either fragments of one vascular cylinder or multiple vascular cylinders. Phloem and other soft tissues separate compartments of the secondary xylem. Compartments of separated xylem tissue can include fragments of i) only adult wood, or ii) adult *and* juvenile wood; either of which can also include areas of separated pith. These vascular variant types are generally derived from multiple cambia and a combination of irregular cambium activities:

- Dissected xylem (Figure 3, A and B): The wood cylinder is divided into multiple fragments of independent but relatively equivalent sizes, with clusters of soft tissues, dividing completely the wood cylinder and may or may not include the pith. The divisions are centered on the pith. The activity of the cambia is either regular, creating an approximatively homogeneous outline of the clusters (Figure 3, A), or irregular, producing deeply grooved outlines for the wood clusters (Figure 3, B).
- Successive cambia (Figure 3, C and D): Cambia arise successively, producing xylem centripetally and phloem centrifugally regularly around the entire girth of the stem, dividing the wood by bands of successive phloem bands (Figure 3, C), or with the presence of axial elements (rays, white lines; Figure 3, D) resulting in phloem head caps in bands and xylem elements wrapped in conjunctive tissue (Figure 3, D).
- Compound (Figure 3, E): The stem is composed of multiple vascular cylinders, these appear in cross-section as irregular multiple woody cylinders each comprising a pith and young and adult wood which are distributed within the cortex or ground tissue of the stem.

Table S2. 164 liana samples, their wood structure categories, vascular variants and additional information concerning their anatomical differences.

Note S2. Vessel delineations of liana stem samples.

Vessel area delineation of different species was compounded by the variable appearance of lumen/wall details visible with the high throughput analysis necessary for handling large numbers of specimens. A common problem was occluded vessel lumens likely resulting from natural causes, tyloses, breakdown of wall material, or artifacts resulting from clogging of the lumens during the sanding and polishing preparations. In a single section, although some vessels might be clear, others were occluded or clogged. Blocked vessels can occur naturally after the production of tyloses, gums, or other deposits, if the vessels are no longer functional. It has been documented that wide liana vessels can be occluded with tyloses or gums as an immediate response to damage and cutting to seal the wounded tissue (Fisher and Ewers 1991). However blocked vessels can also be artifactual after sanding and polishing owing to sawdust particles. Although these technical problems could potentially lead to an overestimation of theoretical water conductance through vessels via the Hagen Poiseuille equation, we elected to delineate all visible entire vessel lumens, whether clogged or not and whether the obstruction was natural or artefactual. We anticipated that this approach would provide a comparable value of conductance, which reflected theoretical water flow for a given state of development, prior to any environmental and/or preparatory effects across all species. Vessel endwall morphology was not characterized in the high-throughput study meaning that putative effects of endwall pitting and intercell membrane architecture could not be evaluated in terms of the calculated maximum conductance. Finally, in some cross-sections, parts of the vascular cylinder appeared to have decayed in otherwise healthy stems. Such areas were not used for vessel area and tissue delimitation, and the samples were excluded from trait analysis.


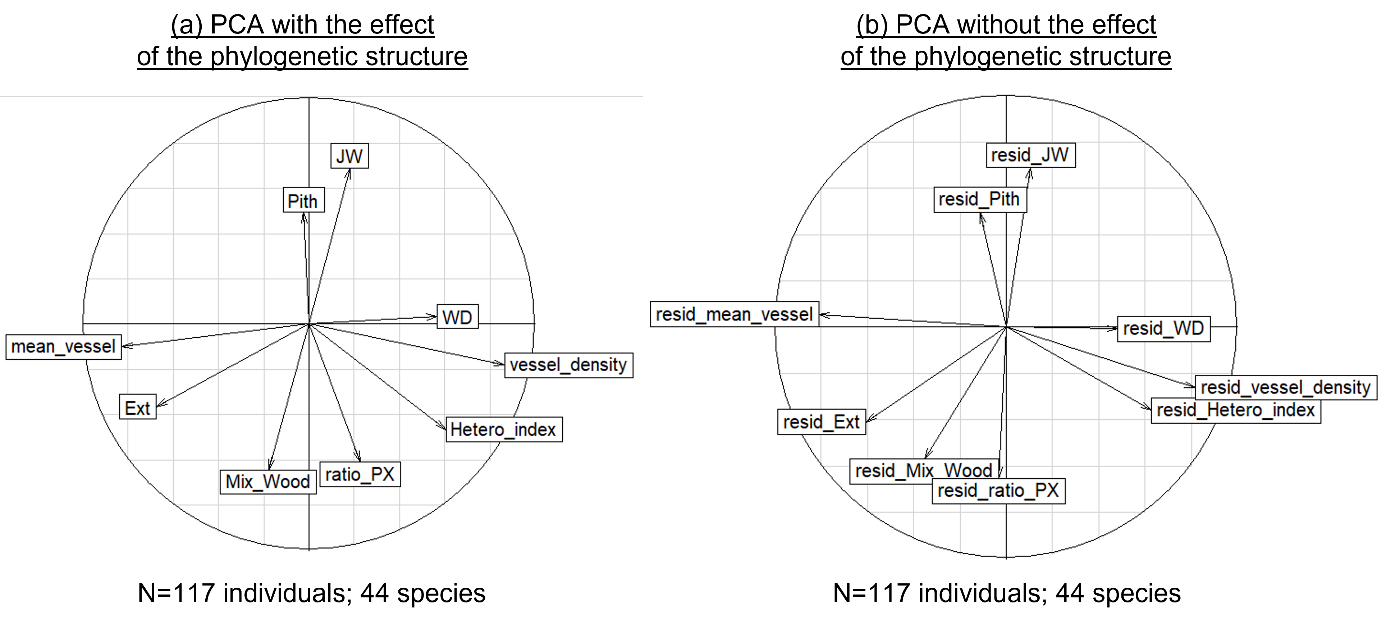


Fig S1. Effect of species identity and phylogenetic structure on trait covariation. Principal Component Analyses (PCA) were performed on anatomical functional traits for a subset of 117 liana individuals for which phylogenetic information was available (44 species). Panel (a) represents a PCA performed on raw trait measurements while panel (b) represent a PCA performed on the residuals of phylogenetic generalized linear mixed models accounting for species identity and phylogenetic structure for all traits (resid_Traits). Pith: pith area; JW: juvenile wood area; Mix_Wood: adult, juvenile and interxylary phloem area; Ext: external tissues; Hetero_index: heterogeneity index; ratio_PX: phloem/xylem ratio; WD: wood density; vessel density: wood vessel density; mean_vessel: mean wood vessel area.


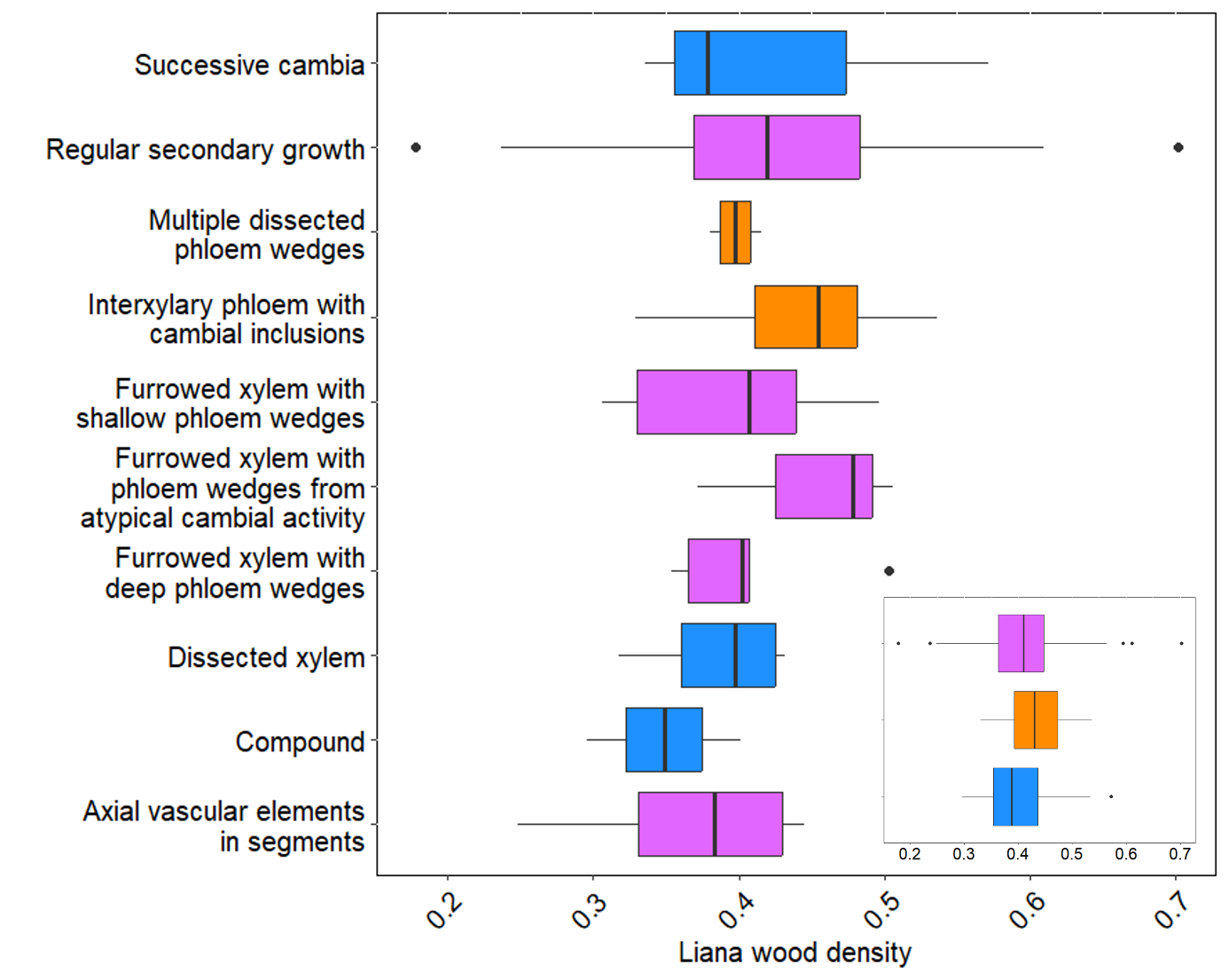


Fig S2. Distribution of liana wood density values across liana vascular variants. Mean values for wood structure categories are represented at the bottom right corner. Uniform, internal phloem and compound wood lianas are in pink, orange and blue respectively.


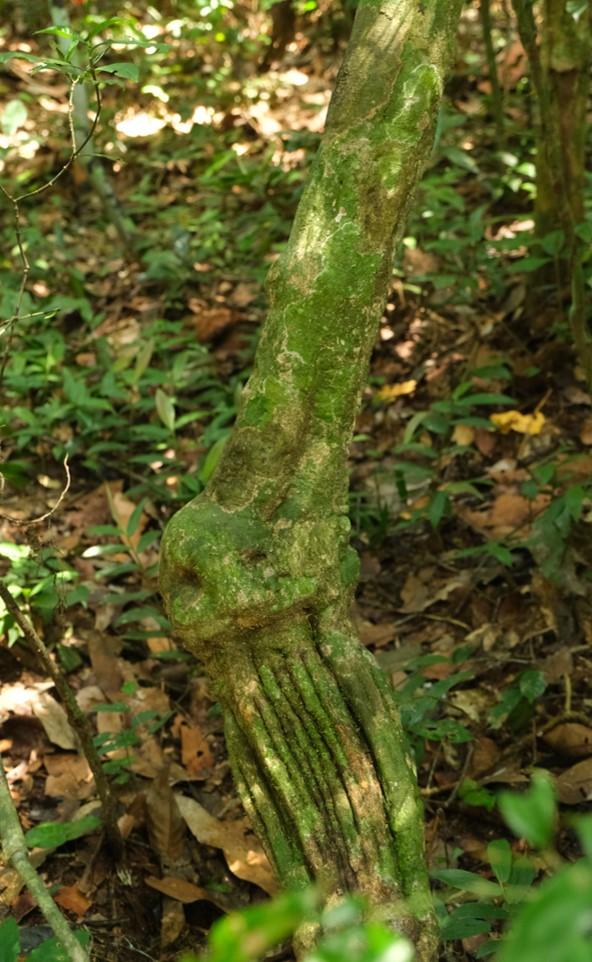


Fig S3. Stem of *Loeseneriella sp.1* (Celastraceae) splitting into several separate strands and reforming as a single stem with scattered wood.


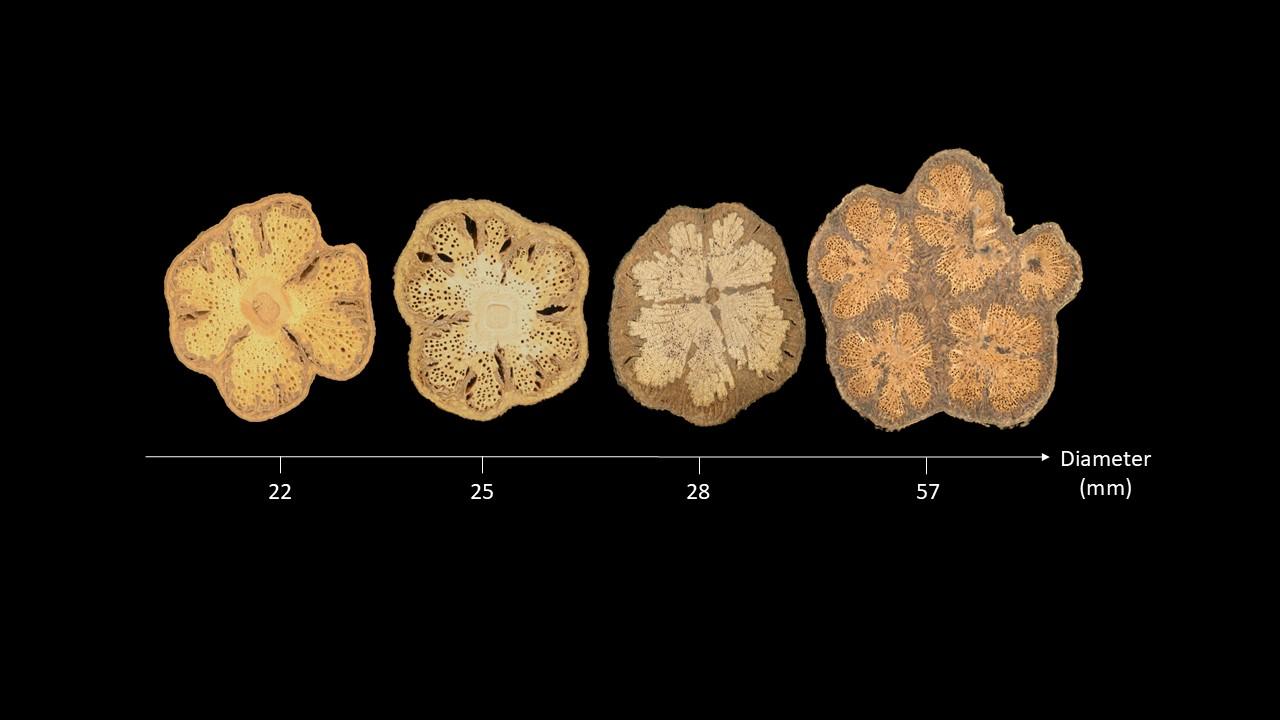


Fig S4. Different vascular variant development in stems of the species *Loeseneriella sp.1* (Celastraceae)*,* with increasing compartmentalization with diameter.
